# KTX207-mediated PDE4D degradation disrupts tumour cell migration, invasion, and angiogenic potential

**DOI:** 10.1186/s12964-026-02946-5

**Published:** 2026-05-21

**Authors:** Alina Zorn, Yi Zhao, Aoife Giblin, Igor Belka, Eduardo Torres, Cathy Swindlehurst, Kyle Chan, David Stirling, George S. Baillie, Yuan Yan Sin

**Affiliations:** 1 School of Cardiovascular and Metabolic Health, University of Glasgow, Glasgow, G12 8QQ UK; 2 Katalytic Therapeutics, 7966 Arjons Drive, Suite D, San Diego, 92126 USA

**Keywords:** PDE4D, PROTAC, Cell migration, Cytoskeletal remodelling, Angiogenesis

## Abstract

**Background:**

Phosphodiesterase 4 (PDE4) enzymes regulate intracellular cyclic adenosine monophosphate (cAMP) and thereby influence multiple cancer-relevant processes. Metastasis and angiogenesis, which rely on coordinated cytoskeletal remodelling and integrin-mediated signalling are key determinants of cancer progression. We previously reported KTX207, a cereblon-based proteolysis-targeting chimera (PROTAC) that selectively degrade PDE4D shortforms and suppresses tumour cell proliferation.

**Methods:**

To further evaluate the therapeutic potential of KTX207, we examined the effects of PDE4D degradation on cancer cell migration, invasion, cytoskeletal organisation, and angiogenic capacity using 2D and 3D models. Assays included wound healing, Boyden chamber invasion, single-cell tracking, 3D spheroid invasion, endothelial tube formation and sprouting assays, as well as immunocytochemistry, Western blotting, and ELISA. Proteasome inhibition was used to assess degradation dependency.

**Results:**

KTX207 markedly impaired A549 cell motility, abrogated directional persistence, disrupted cytoskeletal architecture with mislocalisation of focal adhesion kinase, and reduced invasive capability. Endothelial cells exhibited reduced angiogenic and sprouting potential. KTX207 altered key regulators of focal adhesion and cytoskeletal signalling, including integrin β1, ezrin, RhoA, and phospho‑Src, and reduced angiogenic factors such as VEGF‑A and angiopoietin‑2. Phospho-FAK levels remained unchanged, indicating disruption of spatial rather than global kinase signalling. Importantly, these effects were attenuated by proteasome inhibition, supporting a degradation-dependent mechanism.

**Conclusion:**

These findings highlight a previously underappreciated role for PDE4D shortforms in coordinating cytoskeletal dynamics and tumour-associated angiogenesis. Targeted PDE4D degradation therefore represents a promising therapeutic strategy for limiting metastatic progression.

**Supplementary Information:**

The online version contains supplementary material available at 10.1186/s12964-026-02946-5.

## Introduction

The metastatic cascade represents the most lethal hallmark of cancer, encompassing a highly orchestrated series of events that enable cancer cells to disseminate from the primary tumour and establish secondary growths in distant organs. This process requires initial detachment of tumour cells from the primary lesion, local invasion into surrounding tissues; intravasation into the vasculature or lymphatics; extravasation into secondary sites, and ultimately colonisation of foreign microenvironments [1]. Each of these steps depends on dynamic remodelling of the actin cytoskeleton and precise regulation of integrin-mediated adhesion signalling. Together, these processes provide both the mechanical forces required for motility and the spatially restricted signalling cues that coordinate directional migration [2, 3]. Dysregulated actin dynamics and integrin-FAK signalling are common features of cancer progression and have also been implicated in pathological tissue remodelling, immune evasion and therapeutic resistance [4, 5]. Clinically, treatment failure in non-small cell lung cancer (NSCLC) is frequently attributed to metastatic spread and invasion into adjacent organs [6], underscoring the urgent need for therapeutic strategies that specifically target metastatic mechanisms.

Phosphodiesterase 4D (PDE4D), a cAMP-specific phosphodiesterase, has emerged as a pivotal regulator of cancer-associated signalling pathways, including those governing proliferation, survival, and migration [7]. Aberrant PDE4D expression or activity has been associated with enhanced tumorigenicity, increased metastatic potential and poor patient outcomes in multiple malignancies. For example, suppression of PDE4D in choriocarcinoma cells attenuates migration and invasion by inhibiting epithelial-to-mesenchymal transition (EMT), highlighting its role in metastatic progression beyond proliferation [8]. Moreover, high PDE4D expression has been shown to promote cell invasion in melanoma through interactions with focal adhesion kinase (FAK) and cytoskeletal regulators [9], while pharmacological inhibition of PDE4D has been shown to suppress mTORC1 signalling and restrain tumour growth in pancreatic cancer [10]. Collectively, these findings suggest that PDE4D is a nodal regulator of both oncogenic signalling and metastatic behaviour, positioning it as a promising therapeutic target.

We previously developed KTX207, a cereblon-based proteolysis-targeting chimera (PROTAC) that selectively degrades PDE4D shortforms at sub-nanomolar concentration [11]. Our earlier work demonstrated that KTX207 which is based around the PDE4 inhibitor BI 1,015,550 (hereafter referred to as BI) [12], effectively reduces PDE4D expression and exerts anti-proliferative effect in A549 cells. Importantly, PROTACs offer distinct advantages over conventional inhibitors by eliminating rather than transiently inhibiting the target protein, thereby overcoming limitations related to enzyme redundancy and compensatory feedback that often lead to drug resistance. Mutations in active sites and binding pockets, which frequently reduce the efficacy of conventional inhibitors, represent another mechanism of resistance that can be effectively circumvented by targeted protein degraders. Recent discoveries have expanded the pharmacological landscape of PDE4 regulation beyond inhibition. For instance, Omar et al. [13]. identified small-molecule allosteric activators of PDE4 long isoforms that enhance enzymatic activity by stabilising active conformations. This work underscores the structural and regulatory diversity of PDE4 isoforms and suggests that selective manipulation whether through activation, inhibition or targeted degradation, can yield distinct biological outcomes.

In the present study, we extend our investigation to assess whether KTX207 can modulate cancer cell migration, invasion, and angiogenesis, which are the three fundamental processes underpinning metastatic progression. We show that KTX207-mediated PDE4D degradation impairs these cellular functions and mechanistically disrupts the ezrin-FAK-integrin β1 (ITGB1) signalling axis, a critical hub that coordinates actin cytoskeletal reorganisation and focal adhesion dynamics. This disruption leads to downregulation of multiple cytoskeletal and angiogenic regulators, defective stress fibre assembly, and loss of adhesion signalling, culminating in reduced invasive and angiogenic potential. By linking targeted protein degradation to functional suppression of metastatic traits, our findings highlight the therapeutic potential of PROTAC-based PDE4D targeting as a strategy for halting tumour progression.

## Methods

### Cell culture and compound treatment

Human non-small-cell lung cancer cell line, A549 (ATCC #CCL-185, RRID: CVCL_0023) and human brain microvascular endothelial cell line, HBEC-5i (ATCC #CRL-3245, RRID: CVCL_4D10) were obtained from the American Type Culture Collection (LGC Standards, Teddington, Middlesex, UK). A549 cells were cultured in Dulbecco’s Modified Eagle Medium (DMEM) supplemented with 10% fetal bovine serum (FBS), 2 mM L-glutamine and 1% penicillin–streptomycin, whereas HBEC-5i cells were cultured in DMEM/F12 (Invitrogen, Carlsbad, California, USA) supplemented with 10% FBS, 1% penicillin/streptomycin and 40 µg/ml endothelial growth serum (Millipore, Burlington, Massachusetts, USA). Both cultures were maintained in a humidified incubator at 37 °C under 5% CO_2_. Cells were split at 80% confluence, and medium was replaced every 2–3 days as required. The PDE4D PROTAC, KTX207 and warhead control BI 1,015,550 were provided by Katalytic Therapeutics (San Diego, California, USA). Unless otherwise specified, cells were typically harvested 24 h after treatment. Bortezomib (BTZ) was used as a proteasome inhibitor to assess ubiquitin-proteasome system dependence of PROTAC-mediated effects. For short-term rescue studies (immunocytochemistry and immunoblotting), cells were pre-treated with 100 nM BTZ for 1 h followed by co-treatment with KTX207 during the final 3 h to inhibit proteasome-dependent degradation during early signalling events. For long-term phenotypic assays (A549 3D spheroid invasion and HBEC sprouting assays), 100 nM BTZ was maintained throughout the 24 h treatment period along with KTX207 to ensure sustained proteasome inhibition during development of the phenotype.

### Western blot analysis

Protein extracts were prepared in lysis buffer (25 mM Tris/HCl pH 7.4, 150 mM NaCl, 1% NP-40, 1 mM EDTA, 5% glycerol) with complete EDTA-free protease inhibitor cocktail tablets (Roche, West Sussex, UK). Equal amounts of protein were separated by SDS-PAGE (4–12% Bis-Tris gels) and transferred onto nitrocellulose membranes. The membranes were blocked in Intercept (TBS) Blocking Buffer (LI-COR Biosciences, Lincoln, Nebraska, USA) for 1 h at room temperature, followed by incubation with primary antibodies diluted in Intercept T20 (TBS) Antibody Diluent (LI-COR Biosciences, Lincoln, Nebraska, USA) overnight at 4 °C with gentle shaking. Primary antibodies used were rabbit anti-PDE4D (#ab171749, 1:3000; Abcam, Cambridge, UK), mouse anti-ezrin (#sc-753, 1:1000; Santa Cruz Biotechnology, Dallas, Texas, USA), mouse anti-RhoA (#sc-418, 1:1000; Santa Cruz Biotechnology, Dallas, Texas, USA), rabbit anti-FAK (#sc-558, 1:50; Santa Cruz Biotechnology, Dallas, Texas, USA), mouse anti-phospho-FAK (Y397) (#05-1140, 1:500; Sigma-Aldrich, Gillingham, UK), mouse anti-Src (#05-184, 1:3000; Upstate, Lake Placid, NY, USA), rabbit anti-phospho-Src (Y416) (#2101, 1:1000; Cell Signaling Technology, Beverly, MA, USA), rabbit anti-E-cadherin (#4065, 1:1000; Cell Signaling Technology, Beverly, MA, USA), rabbit anti-ZEB1 (#ab203829, 1:500; Abcam, Cambridge, UK). Mouse anti-GAPDH (# 60004-1-Ig, 1:80 000), rabbit anti-integrin β1 (ITGB1) (#12594-1-AP, 1:1000), rabbit anti-vascular endothelial growth factor A (VEGF-A) (#19003-1-AP, 1:5000), rabbit anti-angiopoietin-2 (ANGPT2) (#24613-1-AP, 1:2000), rabbit anti-N-cadherin (#22018-1-AP, 1:10 000), rabbit anti-SNAI1 (#13099-1-AP, 1:500), rabbit anti-TWIST1 (#25465-1-AP, 1:1000), and rabbit anti-vimentin (#60330-1-Ig, 1:5000) were from Proteintech (Manchester, UK). Membranes were washed with TBST and then incubated with fluorescently labelled secondary antibodies for 1 h at room temperature. The secondary antibodies used were IRDye 680RD donkey anti-mouse IgG (#926 − 68 072, 1:20000) and IRDye 800CW donkey anti-rabbit IgG (#926 − 32 213, 1:20000) from LI-COR Biosciences (Lincoln, Nebraska, USA). Images were acquired using the Li-Cor Odyssey CLx Imaging System with signals detected in the 700 and 800 nm channels. All densitometry analyses were performed using LI-COR Image Studio and normalised to GAPDH. Representative images are shown in grayscale.

### Wound healing assay

A549 cells were seeded in 6-well plates and grown to confluence. The cell monolayer was scratched in a straight line using a sterile P200 micropipette tip and the wells were then carefully washed with PBS to remove debris and non-adherent cells to prevent reattachment in the wound area. Cells were then treated and cultured in medium containing reduced FBS (2%) to minimise proliferation and ensure that scratch closure reflected cell migration. Images were captured immediately after wounding (≥ 5 independent fields per well) and at the same location after 8 h and 21 h of incubation using a DS-Fi3 camera with NIS Elements software (Nikon). Wound areas were measured using ImageJ plugin ‘MRI Wound Healing Tool’ (NIH, Bethesda, MD, USA) by outlining the open gap. Wound closure was expressed as the percentage (%) change in wound area at the indicated time point (8–21 h) relative to the initial wound area at 0 h.

### Boyden chamber invasion assay

Cell invasion was assessed using a 48-well Boyden chamber (Neuro Probe Inc, #AP48; Gaithersbury, Maryland, USA) with a collagen IV-precoated (10 µg/ml) polycarbonate membrane (8 μm pore size, 25 × 80 mm) according to the manufacturer’s protocol. Briefly, cells were seeded in the upper chamber at a density of 5 × 10^4^ cells per well in 50 µl medium containing 1% FBS and treatment. The bottom chamber contained 25 µl standard medium with 10% FBS. Serum-free medium was added to three wells as a negative control. After 8 h of incubation at 37 °C with 5% CO_2_, non-invading cells were removed from the apical surface of the porous membrane with a moistened cotton swab. Invading cells that had passed through the collagen IV barrier on the underside were fixed with 4% (v/v) paraformaldehyde for 15 min at room temperature and stained with 0.25% crystal violet in 20% methanol for 20 min. The membranes were then washed in Milli-Q water and photographed.

### Single-cell tracking assay

To analyse individual cell movement, A549 cells were seeded sparsely at 1.2 × 10^4^ cells in 24-well plates and grown overnight at 37 °C with 5% CO_2_. Treatments were initiated the next day and cell motility was immediately monitored using a Nikon AX-R microscope equipped with temperature- and CO_2_-controlled environment. Time-lapse images were acquired every 20 min for 18 h using a 20x objective. Individual cell trajectories were tracked manually using the ImageJ Manual Tracking plugin (NIH, Bethesda, MD, USA), and migration parameters were analysed using the Chemotaxis and Migration Tool (Ibidi, GmbH, Martinsried, Germany). Parameters included velocity, directness, accumulated distance (the total path length that cell travelled) and Euclidian distance (the straight-line distance between the starting and ending point of the cell). Directionality was defined as the ratio of Euclidean distance over accumulated distance. Mean velocity was calculated as accumulated distance divided by total tracking time. Only non-dividing cells were analysed.

### Alamar Blue assay

Alamar Blue (resazurin) assay was performed to evaluate treatment-induced cytotoxicity. A549 cells were seeded into Greiner 96-well black plate with flat clear bottom at 1 × 10^4^ cells per well and cultured in phenol red-free medium for 24 h prior to the assay. Cells were then treated with the indicated treatments for 24 h. Following treatment, Alamar Blue cell viability reagent (#A50100, Invitrogen, Eugene, OR, USA) was added directly to each well at 10% (v/v) of the culture volume and incubated for 2 h at 37 °C in a humidified atmosphere containing 5% CO_2_. Metabolically active cells convert resazurin to resorufin, a red-fluorescent indicator, thereby generating a quantitative measure of viability and cytotoxicity. Fluorescence was measured using a Berthold Tristar 5 microplate reader (Baden-Württemberg, Germany) at an excitation wavelength of 560 nm and an emission wavelength of 595 nm. Background fluorescence from media-only wells was subtracted. Doxorubicin (1 µM) was used as a positive control to confirm assay sensitivity to cytotoxic effects. Data were normalised to untreated controls and expressed as relative fluorescence intensity (%). All experiments were performed with four technical replicates and repeated in six independent biological experiments.

### Tube formation assay

On the day of the assay, 10 µl of ice cold Geltrex (Gibco #A1413201) was added to the inner well of a µ-Slide angiogenesis (Ibidi, Germany) and incubated for 1 h at 37 °C with 5% CO_2_. During the incubation time, HBEC cells were harvested and counted. 50 µl of cell suspension containing 1.2 × 10^4^ cells in conditioned media collected from treated or control A549 cells, was seeded onto each well containing the polymerised Geltrex. Cells were allowed to form tubular networks for 8 h before visualising on an inverted phase-contrast microscope at 10x magnification (Eclipse Ts2, Nikon Corporation). Five images per well (centre of the well and four cardinal points) were captured at 21 h using a DS-Fi3 camera with NIS Elements software (Nikon). For automated quantification of network formation, images were analysed using ImageJ plugin ‘Angiogenesis Analyzer’ (NIH, Bethesda, MD, USA) [14]. Parameters including the number of junctions, total tube length, number of meshes, and total mesh area were analysed as a measurement of tube forming ability.

### Enzyme linked immunosorbent assay (ELISA)

Culture supernatants from A549 cells following 24 h of the indicated treatments (in phenol red-free medium used as conditioned medium for tube formation assay) were collected from 12-well plates and stored at -20 °C until assayed. VEGF levels were measured using the Human VEGF ELISA Kit (#ab222510, Abcam, Cambridge, UK) according to the manufacturer’s instructions. Briefly, cell culture supernatants were centrifuged at 2000 x g to remove debris. Supernatants were diluted in Sample Diluent NS with 2X Enhancer and added to the appropriate wells of the microplate strips (50 µl/well), followed by incubation with 50 µl Antibody Cocktail for 1 h at room temperature on a plate shaker set to 400 rpm. Wells were then washed three times with Wash Buffer PT before incubation with 100 µl TMB Substrate for 10 min in the dark. The reaction was stopped by adding 100 µl Stop Solution and absorbance was measured at 450 nm using a Berthold Tristar 5 microplate reader (Baden-Württemberg, Germany). VEGF concentrations were determined by comparison with a standard curve generated using VEGF standards.

### HBEC spheroid-based sprouting assay

Due to unavailability of human umbilical vein endothelial cells (HUVECs), HBEC-5i cells were used as an alternative endothelial model for sprouting analysis. Briefly, 100 µl of complete medium containing 1000 HBEC cells was seeded into each well of a NunclonTM SpheraTM 96-well U-shaped-bottom microplate (#174925, Thermo Fisher Scientific, Roskilde, Denmark). Plates were centrifuged at 1500 rpm for 10 min to promote cell aggregation and spheroids were allowed to form over 48 h. Individual spheroids were embedded in 30 µl of 2.5 mg/ml rat tail collagen 1 (#A1048301, Gibco, Waltham, MA, USA) diluted in cold medium containing 1% FBS and transferred to 24-well plates (one spheroid per well). The gels were polymerised at 37 °C for 1 h. Following gelation, collagen-embedded spheroids were overlaid with 200 µl warm medium containing 2% FBS to minimise proliferation. To ensure a robust angiogenic response using HBEC-5i spheroids, exogenous human VEGF165 recombinant protein (25 ng/ml) (#HZ-1038, Proteintech, Manchester, UK) was added to promote sprouting ability, along with the indicated treatments for 24 h. Ten-fold higher concentration of KTX207 and BI was used for spheroids as described previously [11]. Sprouting was imaged after 24 h using an inverted phase-contrast microscope at 10x magnification (Nikon Eclipse Ts2) equipped with a DS-Fi3 camera and NIS Elements software (Nikon). Images were processed in ImageJ (NIH, Bethesda, MD, USA) by converting to 8-bit and applying the Triangle thresholding method to define the spheroid boundary (mask). Sprouts were manually counted and used as readout for the angiogenic activity. A sprout was defined as a multicellular projection extending from the spheroid body exceeding 10 μm in length. At least five spheroids per condition were analysed per experiment and experiments were independently repeated five times.

### Actin cytoskeleton staining

Following treatment, A549 cells grown on chamber slides were fixed with 4% (v/v) paraformaldehyde for 15 min at room temperature and permeabilised with 0.1% (v/v) Triton X-100 in PBS for 10 min. After three washes with PBS, cells were blocked with 10% donkey serum and 2% BSA (w/v) in TBS for 2 h followed by three washes with PBS. Cells were incubated overnight with rabbit anti-FAK (#sc-558, 1:50; Santa Cruz Biotechnology, Dallas, Texas, USA) at 4 °C. After washing with PBS, cells were incubated with Alexa Fluor 488 donkey anti-rabbit IgG (#A21206, 1:500; Invitrogen, Carlsbad, California, USA) for 1 h, followed by F-actin staining with CoraLite 594-conjugated phalloidin (#FP00003, 1:400; Proteintech, Manchester, UK) for 30 min at room temperature. Cells were washed and mounted using ProLong Gold Antifade Mountant with DAPI (Invitrogen, Carlsbad, California, USA). Images were acquired using an upright Zeiss LSM 880 confocal laser scanning microscope controlled with the Zeiss ZEN imaging software, using a Plan-Apochromat 63x / 1.40 oil DIC M27 objective in frame scan mode. Maximum intensity projections (MIPs) were generated from 11 consecutive z-Sect.  (5 μm). F-actin stress fibres were quantified using a custom detection algorithm implemented in the MATLAB Imaging Toolkit (NovoMedix, USA), which combined edge detection, circularity analysis, dynamic intensity thresholding, and filament ratio dilation to accurately identify and quantify the pixel area occupied by stress fibres in each image. Quantification was performed for all experimental conditions, and representative images were used to illustrate qualitative differences in stress fibre organisation.

### 3D spheroid invasion assay

Two hundred microlitres of complete medium containing 3750 A549 cells were seeded into each well of a Nunclon™ Sphera™ 96-well U-shaped-bottom microplate (#174925, Thermo Fisher Scientific, Roskilde, Denmark). Plates were centrifuged at 1500 rpm for 10 min to promote cell aggregation and cells were allowed to form spheroids for 72 h. Individual spheroids were then transferred to a 24-well plate (one spheroid per well) and embedded in 30 µl of 1.25 mg/ml rat tail collagen 1 (#A1048301, Gibco, Waltham, MA, USA) prepared in cold medium containing 2% FBS. Collagen gels were allowed to polymerise at 37 °C for 1 h. Following gelation, collagen-embedded spheroids were overlaid with 200 µl warm medium containing 2% FBS to minimise proliferation, supplemented with 5 ng/ml human transforming growth factor‑beta (TGF-β) (#HZ-1011, Proteintech, Manchester, UK) to promote invasion, and the indicated treatments. Ten-fold higher concentration of KTX207 and BI was used for spheroids as described previously [11]. For rescue experiments, 100 nM bortezomib was maintained throughout the 24 h treatment period along with KTX207. Spheroids were visualised on an inverted phase-contrast microscope at 4x magnification (Nikon Eclipse Ts2), and images were acquired using a DS-Fi3 camera with NIS Elements software (Nikon) at 1 h and 24 h after collagen embedding. A549 spheroid invasive capacity was quantified by measuring spheroid area using ImageJ software (NIH, Bethesda, MD, USA). Invasion was expressed as the percentage (%) change in spheroid area at 24 h relative to the initial spheroid area at 1 h following embedding into the 3D matrix.

### Statistical analysis

All statistical analyses were performed using GraphPad Prism 8 software. Data are presented as mean ± SEM from at least five independent experiments. For initial exploratory experiments, statistical significance was assessed using one-way ANOVA followed by Tukey’s post hoc test for multiple comparisons. For later mechanistic experiments evaluating PROTAC inhibition and rescue, one‑way ANOVA with Sidak’s pre‑selected pairwise comparisons was used to test specific hypotheses. Preplanned comparisons were conducted between KTX207 vs. DMSO (PROTAC effect), KTX207 vs. BI (specificity), KTX207 vs. KTX207 + BTZ (rescue), DMSO vs. BTZ (BTZ interference). A p-value of < 0.05 was considered statistically significant.

## Results

### KTX207 suppresses collective cell migration and invasion

**Fig. 1:**
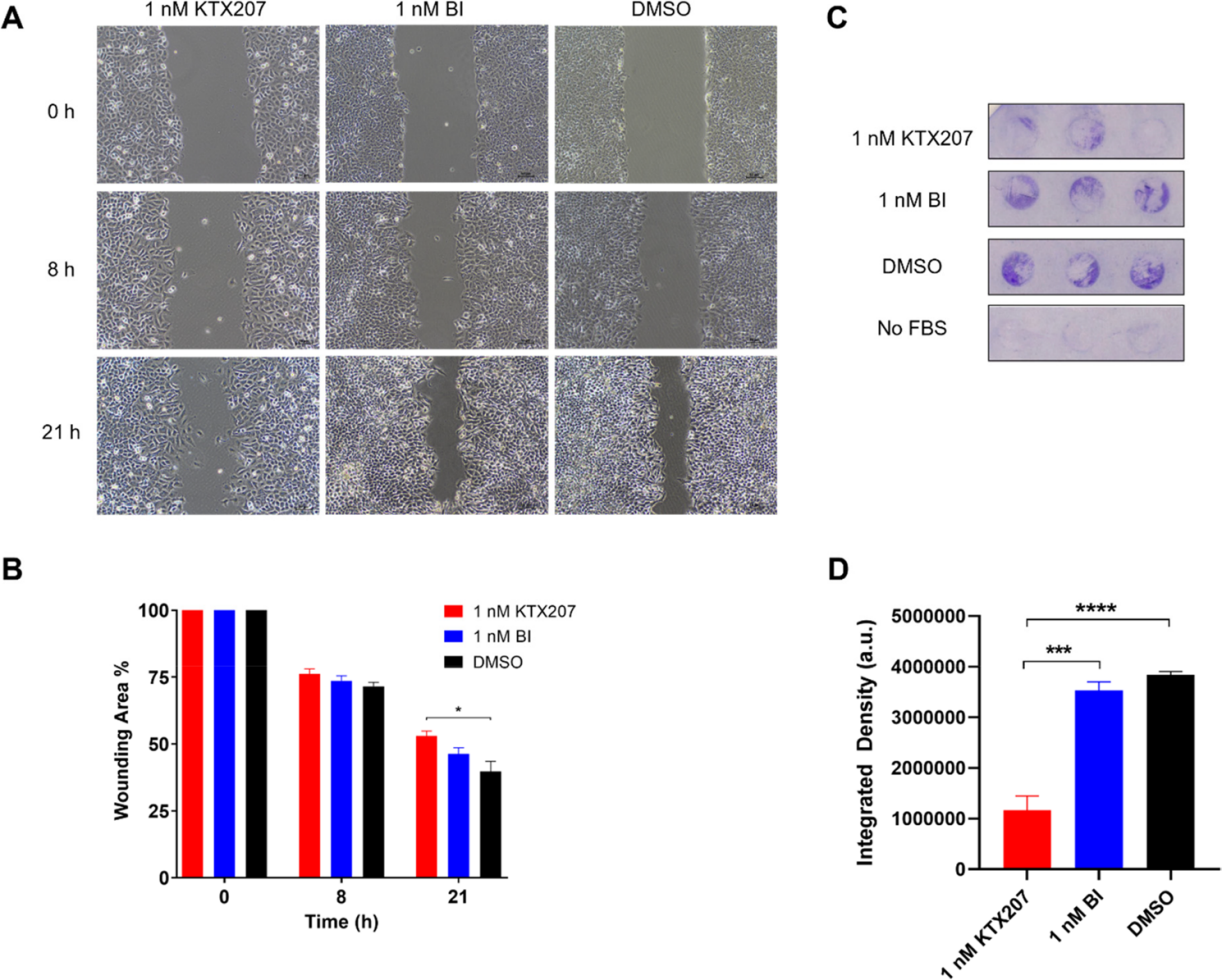
Analysis of A549 cell migration and invasion assays. **A** Microscopy images of wound closure at different treatment conditions at 0 h, 8 h and 21 h after scratching. **B** Quantification of the wounded area invaded during the 21 h presented as percentage normalised to the total area of the wound at 0 h. Scale bar = 50 μm. **C**-**D** Boyden chamber invasive assay. Serum-free medium was used as the negative control for the assay. Cells that passed through the collagen IV barrier were stained with crystal violet. The representative images and intensity index of invaded cells after being stained with 0.25% crystal violet. Data are presented as mean ± SEM (*n* = 5-6). All statistical differences were examined by one-way ANOVA followed by Tukey’s post hoc test for multiple comparisons. *P < 0.05, **P < 0.01, ***P < 0.001, ****P < 0.0001

Given that tumour development and progression are largely dependent on cell motility, we performed wound healing assays to assess the effect of KTX207 on the migratory ability of A549 cell monolayers. After 21 h, treatment with 1 nM KTX207 significantly delayed wound closure, resulting in only partial closure and less gap filling compared to the DMSO-treated control which achieved nearly 65% closure, whereas BI-treated cells (∼55% closure) showed no significant difference (Fig. 1A-B). Similarly, Boyden chamber invasion assays which measure the propensity of A549 cells to spread, showed a marked suppression of invasive capacity, with up to an 75% reduction in the number of invaded cells following treatment with 1 nM KTX207 (Fig. 1C-D) compared to BI and DMSO control.

### KTX207 alters single-cell migration dynamics and does not affect cell viability

**Fig. 2:**
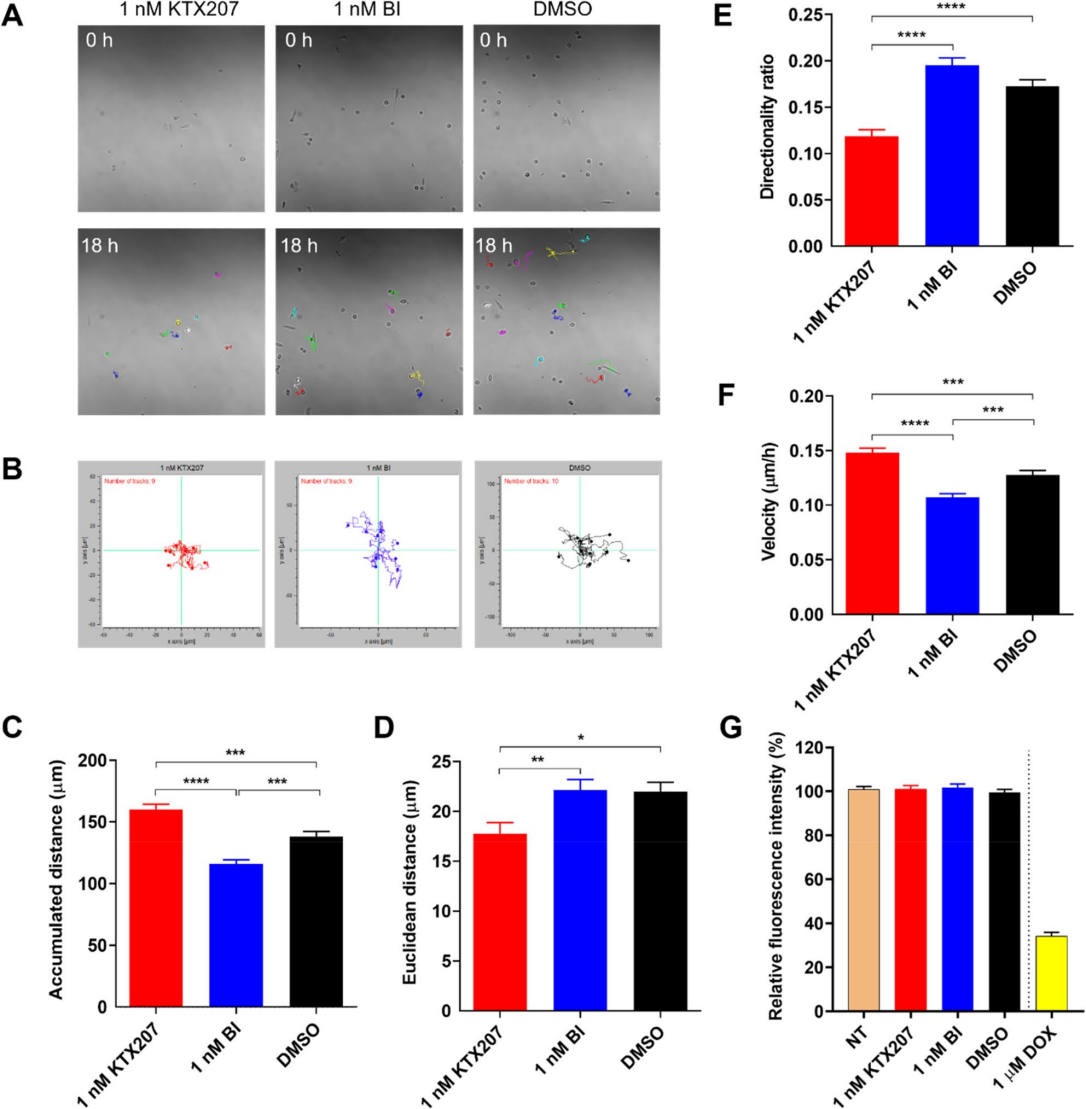
Cell migration evaluation by time-lapse microscopy and single-cell tracking analysis. **A** Representative brightfield images of A549 cells with different treatment conditions captured by time-lapse microscopy showing cell trajectory tracks marked in different colours. **B** Plots showing individual cell trajectories plotted from a common origin (intersection of x and y axes) using Chemotaxis and Migration plugin. Quantitative analysis of (**C**) accumulated distance, (**D**) Euclidean distance, (**E**) directionally ratio and (**F**) velocity (µm/h). 140 cells were analysed for each group. Data are presented as mean ± SEM (*n* = 5). **G** Alamar Blue cell viability assay. The graph illustrates the fluorescence levels of resorufin in cells after 24 h treatment. The amount of fluorescence is proportional to the number of living cells and corresponds to the cell’s metabolic activity. Doxorubicin (1 µM) was used as a positive control. Data were normalised to untreated control and expressed as relative fluorescence intensity (%). Data are presented as mean ± SEM (*n* = 6). All statistical differences were examined by one-way ANOVA followed by Tukey’s post hoc test for multiple comparisons. *P < 0.05, **P < 0.01, ***P < 0.001, ****P < 0.0001. NT, no treatment. DOX, doxorubicin

As cancer cells often migrate following cytoskeletal activity during early invasion and metastasis [2], we next examined A549 cell migration at the single-cell level. We performed live-cell time-lapse microscopy followed by single-cell tracking to monitor longitudinal drug responses (Fig. 2A). Cell trajectories were plotted by extracting the time-series coordinates of the nuclei and resetting them to the origin (0,0). As shown in Fig. 2B, the trajectories of KTX207-treated cells were more confined compared with extended paths in BI and DMSO control, indicating restricted migration. Although KTX207-treated cells retained motility, their movement was less directional, as reflected by reduced Euclidean distance and directionality ratio despite increased instantaneous velocity (Fig. 2C-F). To determine whether the observed phenotypic effects of KTX207 were influenced by cytotoxicity, cell viability was assessed using the Alamar Blue assay. No significant reduction in viability was observed under the conditions used (Fig. 2G), suggesting that the observed phenotypic changes are unlikely to be secondary to cytotoxic stress and instead reflect specific functional alterations.

### KTX207 reduces VEGF secretion and impairs angiogenic potential

**Fig. 3:**
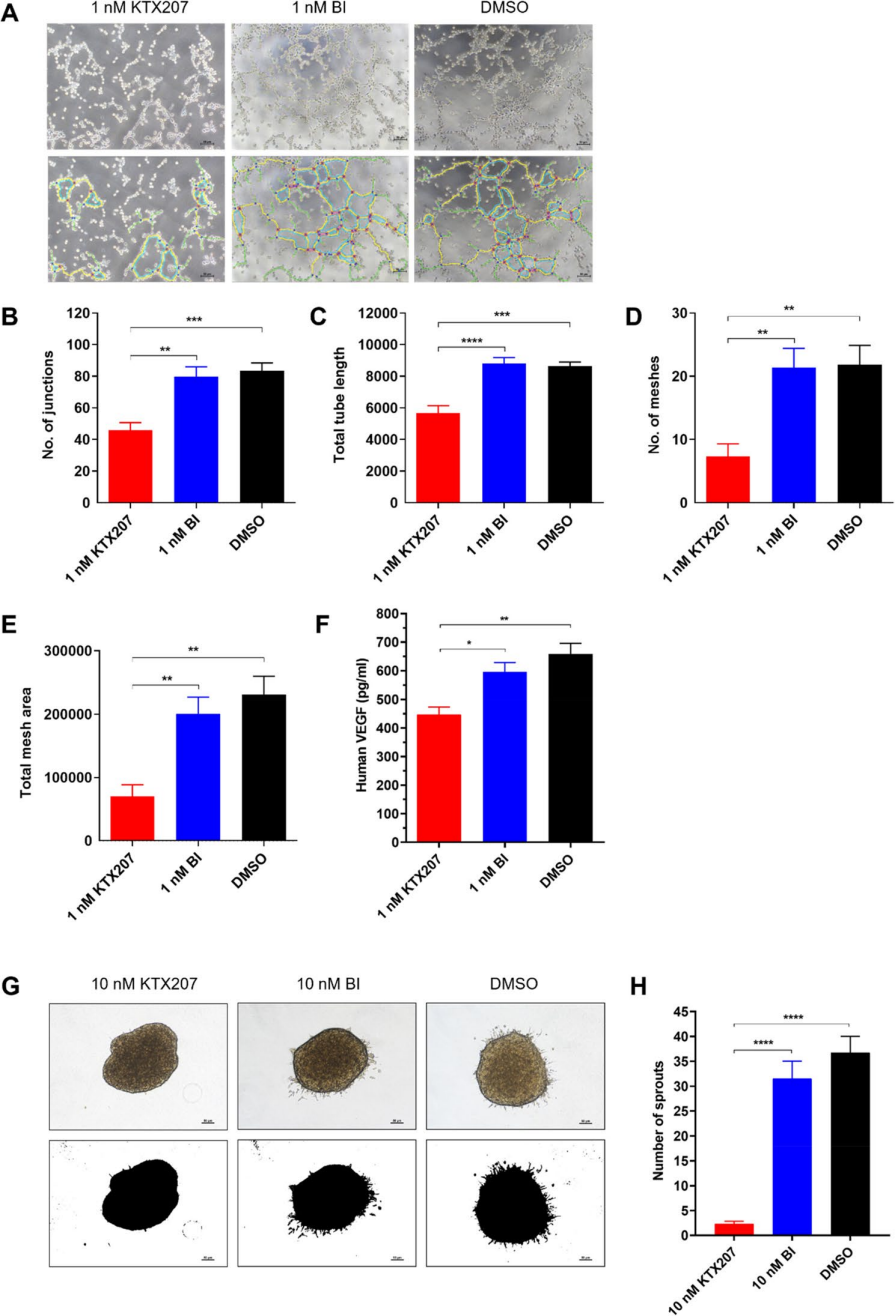
KTX207 impairs endothelial tube formation, reduces VEGF secretion and spheroid sprouting. **A** Top: Representative phase contrast images at 10x magnification of HBEC-5i tube formation stimulated with conditioned medium from A549 cells treated for 24 h with 1 nM KTX207, 1 nM BI or DMSO. Scale bar = 50 μm. Bottom: Summary of the vectorised objects superimposed to the initial images obtained from ImageJ *“Angiogenesis Analyser”* analysis showing loops (yellow), branches (green), junctions (red circle) and meshes (cyan). **B**-**E** Quantification of the angiogenic parameters at 21 h, including number of junctions, total tube length, number of meshes, and total mesh area. **F** VEGF levels in conditioned medium measured by ELISA. **G** Representative images of HBEC-5i spheroid sprouting after 24 h of treatment. Spheroids were cultured in low FBS (1%) medium stimulated with VEGF (25 ng/ml). Top panel: Raw images of collagen-embedded spheroids. Bottom panel: Binary masks generated using Triangle thresholding method for sprout quantification. **H** Quantification of sprouts longer than 10 μm. At least five spheroids per condition per experiment were analysed. Data are presented as mean ± SEM (*n* = 5). Statistical analysis was performed using one-way ANOVA followed by Tukey’s post hoc test for multiple comparisons. *p < 0.05, **P < 0.01, ***P < 0.001, ****P < 0.0001. VEGF, vascular endothelial growth factor

Tumour angiogenesis supplies oxygen and nutrients to cancer cells and facilitates metastasis [1]. Tube formation assays were therefore performed to evaluate the effect of KTX207 on tumour-driver angiogenesis using HBEC-5i cells cultured in conditioned medium derived from treated A549 cells. Defined tube-like structures became visible in BI and DMSO controls after 21 h of seeding. In contrast, KTX207-treated cells remained as isolated clumps and failed to form an interconnected tube network (Fig. 3A). Morphometric analysis focused on length- and area-based parameters, including the number of junctions, total tube length, number of meshes and total mesh area to quantify endothelial sprouting and tubular formation. Quantitative analysis showed that all four parameters were significantly lower in KTX207-treated cells than in BI and DMSO control, indicating impaired angiogenic capacity (Fig. 3B-E). Given the role of VEGF as a key pro-angiogenic factor in cancer-induced angiogenesis [1], VEGF levels in the conditioned medium were measured by ELISA to determine whether the reduced angiogenic activity observed in tube formation assays was associated with altered secretion of pro-angiogenic factors such as VEGF. Consistent with its inhibitory effect on tube formation, KTX207-treated cells exhibited lower VEGF levels compared to BI- and DMSO-treated controls (Fig. 3F), suggesting that KTX207 suppresses angiogenic signalling, at least in part, through reduced VEGF availability. The impact of KTX207 on endothelial behaviour was further assessed using a 3D HBEC-5i spheroid sprouting assay in the presence of exogenous VEGF (25 ng/ml). Under these pro-angiogenic conditions, KTX207 significantly impaired sprout formation compared to DMSO and BI, indicating suppression of endothelial angiogenic capacity despite VEGF stimulation.

### KTX207 alters motility‑associated signalling independently of EMT

**Fig. 4:**
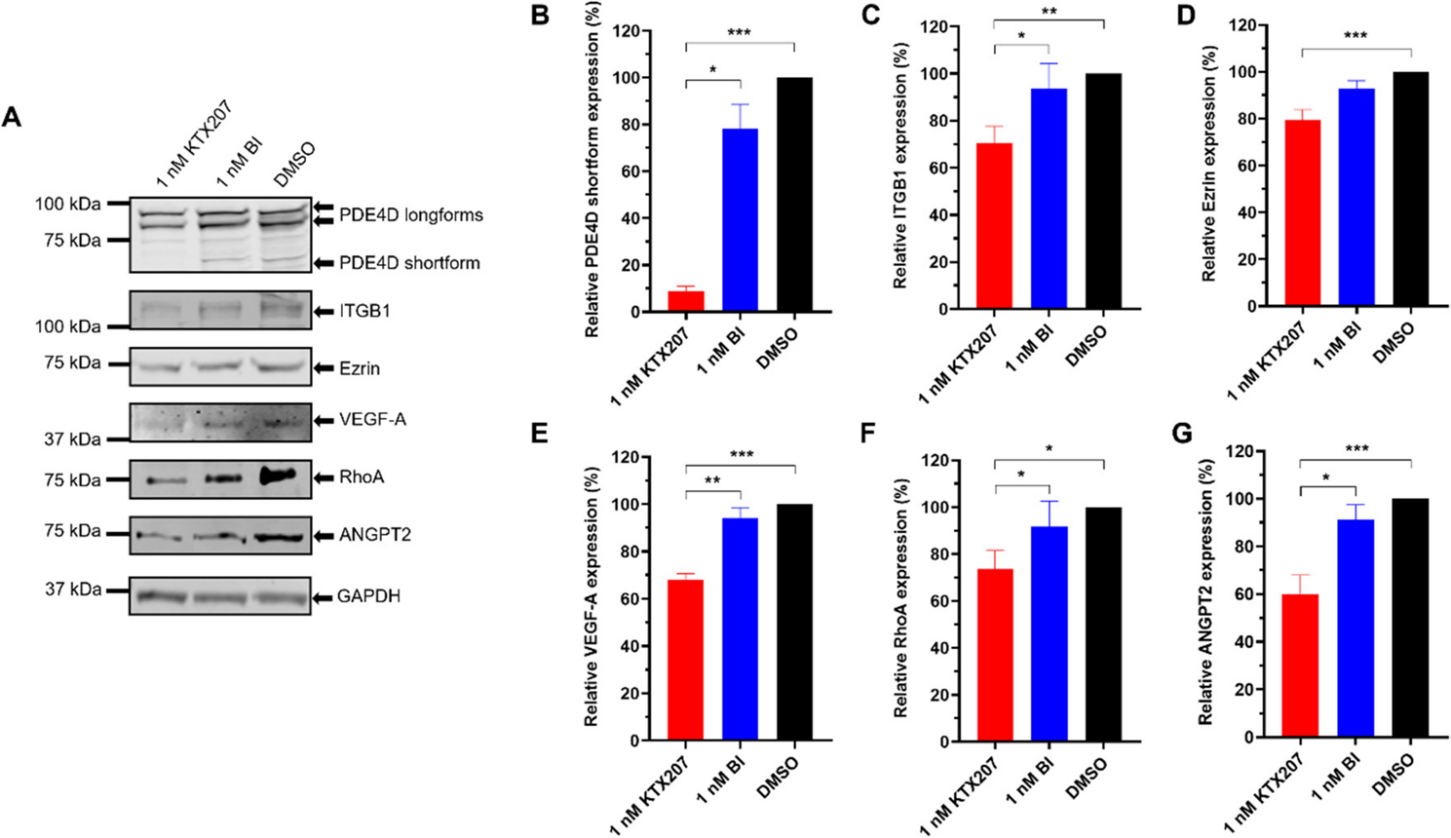
KTX207 downregulates key cytoskeletal and angiogenic proteins. **A** Representative immunoblots showing PDE4D, ITGB1, ezrin, VEGF-A, RhoA and ANGPT2 in A549 cells after 24 h of treatment as indicated. GAPDH was the loading control. **B** -**G** Quantification of the proteins normalised to GAPDH and expressed relative to DMSO (set to 100%). Data are presented as mean ± SEM (*n* = 6). All statistical differences were examined by one-way ANOVA followed by Tukey’s post hoc test for multiple comparisons. *P < 0.05, **P < 0.01, ***P < 0.001, ****P < 0.0001

**Fig. 5:**
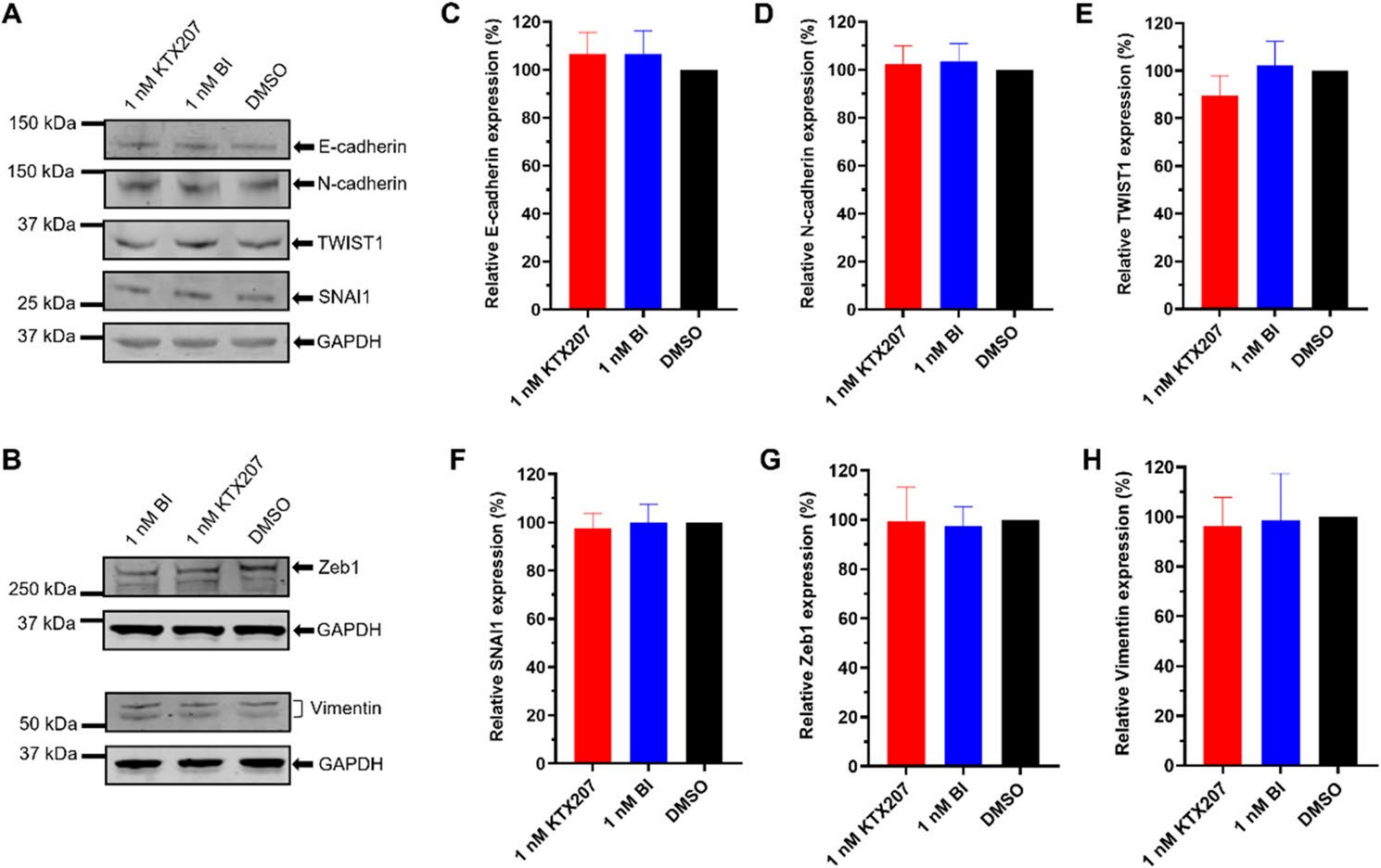
Effect of KTX207 on EMT markers. **A**-**B** Representative immunoblots showing E-cadherin, N-cadherin, TWIST1, SNAI1, Zeb1 and Vimentin in A549 cells after 24 h of treatment as indicated. GAPDH was the loading control. **C**-**H** Quantification of the proteins normalised to GAPDH and expressed relative to DMSO (set to 100%). Data are presented as mean ± SEM (*n* = 6). All statistical differences were examined by one-way ANOVA followed by Tukey’s post hoc test for multiple comparisons

Given the morphologically distinct phenotype of A549 cells following KTX207 treatment, we examined signalling pathways that regulate actin dynamics and angiogenesis. Immunoblot analysis confirmed the reduction in PDE4D shortform protein after KTX207 treatment (Fig. 4). KTX207 also decreased key motility regulators, including ezrin, ITGB1 and RhoA. In parallel, angiogenic factors such as VEGF-A and ANGPT2 were also suppressed by KTX207 (Fig. 4). However, no significant differences were observed in proteins associated with EMT-related pathways, including E-cadherin, N-cadherin, SNAI1, and TWIST1 (Fig. 5). Additional EMT markers, Zeb1 and Vimentin, likewise showed no differences (Fig. 5B and G-H), indicating that the KTX207 does not induce a canonical EMT programme.

### KTX207 disrupts actin cytoskeleton and focal adhesion organisation in a proteasome-dependent manner

**Fig. 6:**
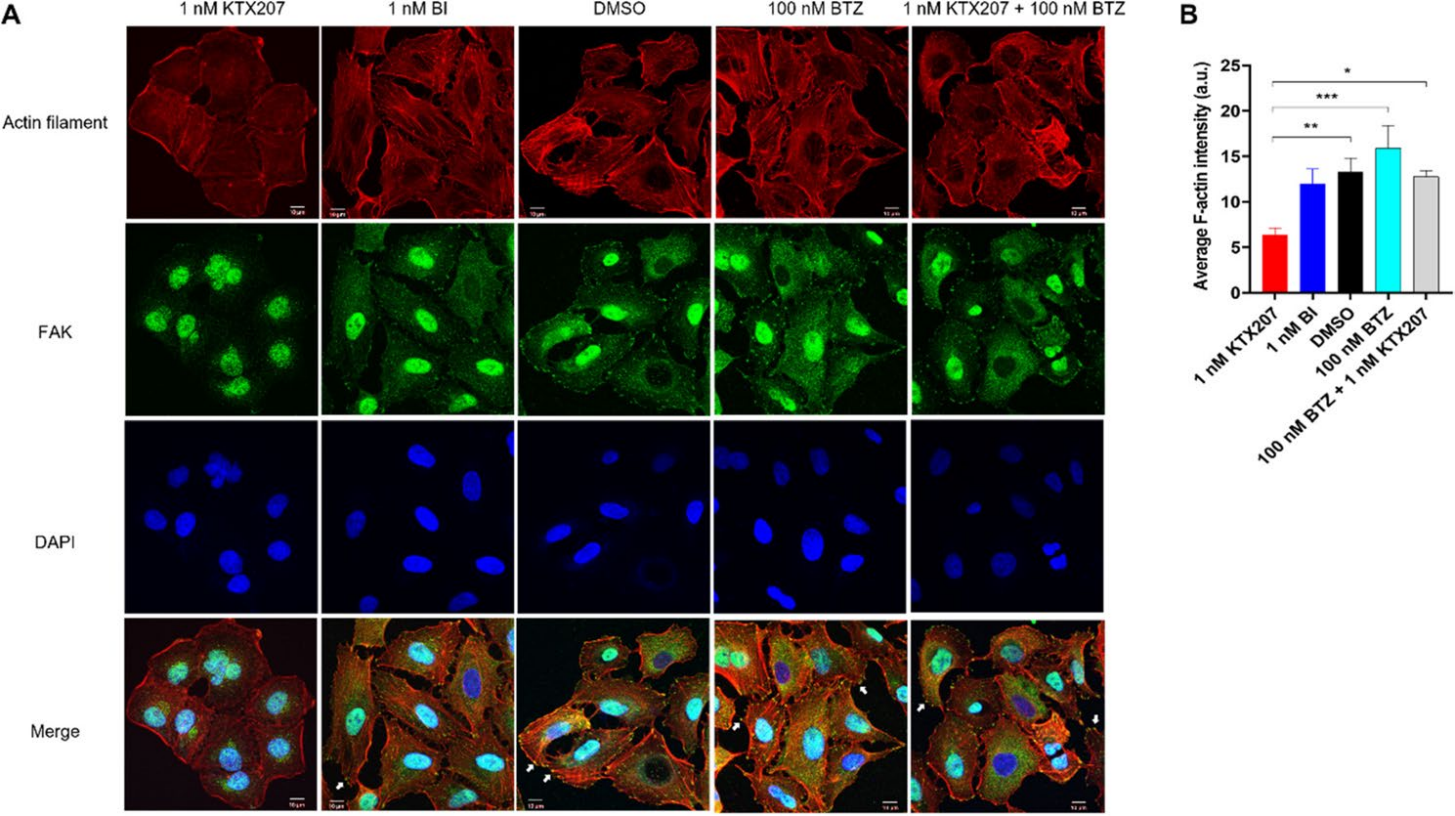
KTX207 disrupts actin filaments and focal adhesions in a proteasome-dependent manner. **A** Representative immunofluorescence images of A549 cells treated with 1 nM KTX207, 1 nM BI, or DMSO for 24 h. For rescue experiments, cells were pre-treated with 100 nM BTZ for 1 h followed by co-treatment with KTX207 for the final 3 h. Actin filaments were stained with phalloidin (red), focal adhesions with anti-FAK antibody (green), and nuclei with DAPI (blue). In DMSO-, BI-, BTZ-treated cells, actin cytoskeletons displayed prominent actin stress fibres and extended morphology, with strong peripheral FAK localisation at focal adhesion sites. In contrast, KTX207 treatment markedly disrupted actin organisation, reducing stress fibre formation and displacing FAK from peripheral adhesion sites. KTX207 co-treatment with BTZ restored actin organisation and peripheral FAK clustering. Scale bars: 10 μm. **B** Quantification of F-actin intensity expressed in arbitrary units (a.u.). The average fluorescence intensity was calculated by dividing the total fluorescence intensity of the entire image by the number of DAPI-stained nuclei. Data are presented as mean ± SEM (n = 5). At least 139 cells were quantified for each condition. Statistical analysis was performed using one-way ANOVA followed by Tukey's post hoc test for multiple comparisons. *P < 0.05, **P < 0.01, ***P < 0.001, ****P < 0.0001. BTZ, bortezomib

Cell motility depends on dynamic restructuring of the actin cytoskeleton. To assess this, we examined actin organisation and FAK localisation in A549 cells by immunofluorescence. KTX207-treated cells exhibited diffuse and granular F-actin staining, whereas BI- and DMSO-treated controls displayed prominent actin stress fibres spanning the cytoplasm and forming cellular protrusions (Fig. 6A). The two different protrusion morphologies, lamellipodia and filopodia are key determinants of migration directionality [15]. In control cells, FAK colocalised with peripheral stress fibres at adhesion sites, reflecting intact focal adhesion-cytoskeleton coupling (white arrows denote representative examples). KTX207 treatment impaired actin remodelling characterised by reduced stress fibres and loss of organised actin architecture (Fig. 6B). KTX207 also caused marked displacement of FAK from the cell periphery, indicating disrupted focal adhesion-cytoskeletal integration. To determine whether these effects were dependent on proteasome-mediated degradation, cells were co-treated with the proteasome inhibitor, BTZ. Co-treatment restored actin organisation and peripheral FAK localisation, with reappearance of stress fibres and FAK clustering. No significant differences were observed among DMSO, BI, BTZ alone, and KTX207 + BTZ groups. Notably, BTZ alone did not significantly differ from DMSO, indicating that the rescue reflects specific reversal of proteasome-mediated KTX207-induced defects rather than independent effects of proteasome inhibition.

### KTX207 suppresses TGF-β-induced invasion in A549 spheroids in a proteasome-dependent manner

**Fig. 7:**
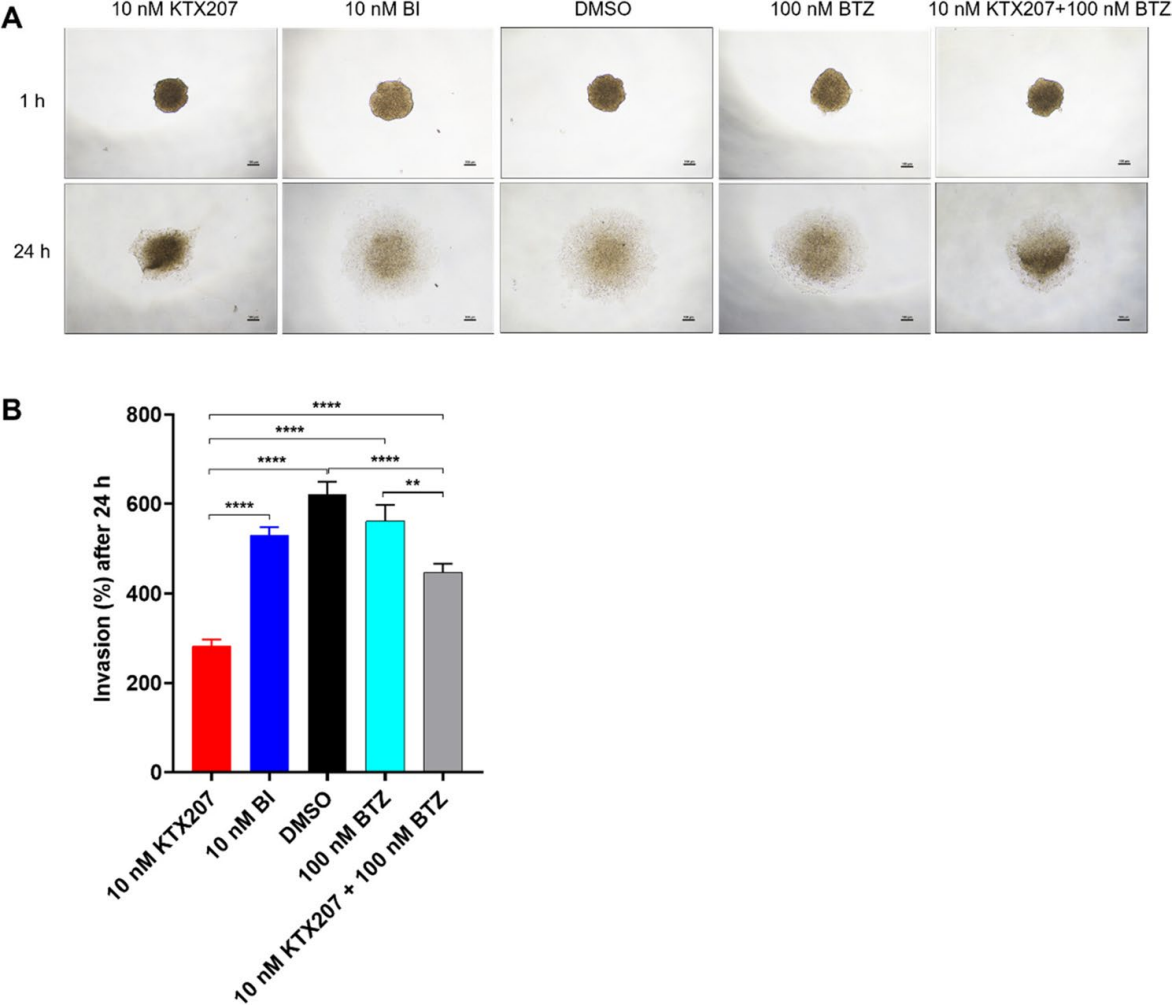
KTX207 suppresses TGF-β-induced A549 spheroid invasion in collagen I matrices. **A** Representative phase-contrast images of A549 spheroids embedded in collagen I matrices and stimulated with TGF-β. Treatments were applied as indicated. Spheroid area was measured at baseline (1 h post-gelation) and after 24 h of treatment. **B** Quantification of spheroid invasion expressed as percentage increase in spheroid area at 24 h relative to the initial spheroid size at 1 h after embedding. Scale bar = 50 μm. Data are presented as mean ± SEM (*n* = 5). A total of 20 spheroids were quantified for each condition. Statistical significance was assessed using one-way ANOVA followed by Tukey’s post hoc test for multiple comparisons. *p < 0.05, **P < 0.01, ***P < 0.001, ****P < 0.0001. BTZ, bortezomib

To evaluate the anti-invasive effects of KTX207 in a physiologically relevant 3D context, we performed spheroid invasion assays using A549 cells embedded in collagen I matrices. Spheroids were stimulated with 5 ng/ml TGF-β to enhance invasive capacity. DMSO- and BI-treated spheroids exhibited pronounced radial invasion into the surrounding collagen matrix, characterised by collective outward invasion from the spheroid core after 24 h (Fig. 7A). In contrast, KTX207 significantly suppressed TGF-β-induced invasion, resulting in minimal expansion beyond the spheroid core. Quantitative analysis revealed a significant reduction in spheroid invasion area in KTX207-treated cells compared with the other groups (Fig. 7B). Co-treatment with BTZ attenuated the inhibitory effect of KTX207, restoring invasive capacity, consistent with a degradation‑dependent mechanism. Importantly, BTZ alone did not significantly alter invasion relative to DMSO, supporting the interpretation that the observed rescue reflects inhibition of KTX207-mediated proteasomal degradation.

### KTX207-mediated PDE4D degradation modulates focal adhesion and cytoskeletal signalling in a proteasome-dependent manner

**Fig. 8:**
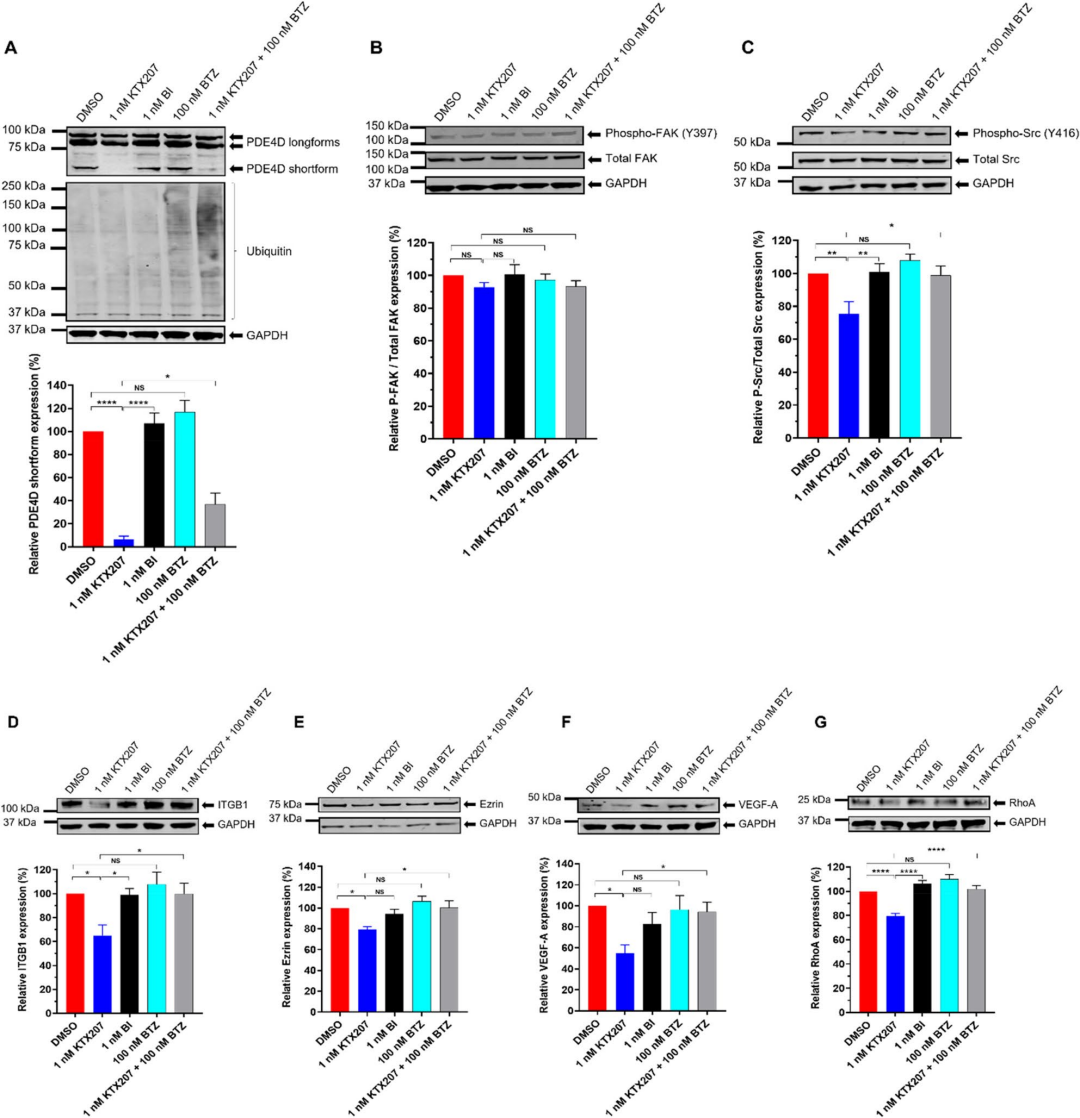
KTX207 downregulates key cytoskeletal and angiogenic proteins in a proteasome-dependent manner. **A**-**G** Top: Representative immunoblots showing PDE4D, phospho-FAK (Y397), phospho-Src (Y416), ITGB1, ezrin, VEGF-A, and RhoA in A549 cells after 24 h of treatment as indicated. GAPDH served as loading control. **A**-**G** Bottom: Quantification of the proteins normalised to GAPDH and expressed relative to DMSO (set to 100%). Data are presented as mean ± SEM (*n* = 5). All statistical differences were examined by one-way ANOVA followed by Sidak’s pre-selected comparisons as indicated. *P < 0.05, **P < 0.01, ***P < 0.001, ****P < 0.0001

To investigate the molecular basis of the cytoskeletal and invasion phenotypes, we examined key regulators of focal adhesion and cytoskeletal signalling by Western blotting. KTX207 treatment markedly reduced PDE4D protein levels. Co-treatment with BTZ attenuated KTX207-induced changes and restored protein levels of several regulators of focal adhesion dynamics, including ezrin, integrin β1, RhoA and VEGF-A (Fig. 8). To assess upstream signalling, we examined phospho-Src (Y416) and phospho-FAK (Y397). KTX207 reduced phospho-Src without altering phospho-FAK, indicating preserved FAK autophosphorylation but impaired Src-dependent signalling (Fig. 8B-C). BTZ alone did not significantly affect protein expression compared with DMSO under the conditions used, indicating that this treatment is well-tolerated and does not substantially affect baseline measurements.

## Discussion

Cell migration is a key determinant of metastatic potential and requires the coordinated integration of actin cytoskeletal remodelling with focal adhesion signalling. FAK is a non-receptor cytoplasmic tyrosine kinase and scaffold protein localised in focal adhesions, where it regulates signals initiated by the integrin family, particularly ITGB1. FAK regulates directional cell movement by orchestrating cytoskeleton organisation, focal adhesions turnover and the formation of membrane protrusions [16]. Integrin clustering recruits FAK to adhesion complexes at the cell periphery, thereby linking the actin cytoskeleton to the extracellular matrix (ECM) [17]. At the leading edge, actin polymerisation drives lamellipodia formation and membrane protrusion, whereas integrin-mediated adhesions stabilise cell-ECM contacts. Together, these coordinate the generation of traction force and enable mechanosensing required for efficient and directional cell movement [2]. Loss of ITGB1 reduces FAK and extracellular signal-regulated kinase (ERK) activation and diminishes migratory capacity [18]. More recently, nanoscale mapping studies have demonstrated that integrin-associated focal adhesion proteins assemble into stratified layers that couple integrin signalling to actin remodelling to regulate force transduction [19].

Cancer cells typically exhibit two distinct modes of motility, i.e. collective and single-cell migration. Standard assays such as wound healing often capture only endpoint outcomes and may be confounded by cell proliferation and cell-cell interactions. To overcome these limitations, we coupled wound healing and Boyden chamber invasion assays with time-lapse microscopy coupled with single-cell tracking analysis to quantitatively assess migratory dynamics. KTX207-treated cells displayed a “fast-but-undirected” migration phenotype, characterised by increased instantaneous motility but reduced directional persistence, reflected in significantly lower Euclidean to accumulated distance ratios. This dissociation between speed and directionality indicates a loss of front–rear polarity and stable leading-edge formation which are critical for productive migration and ECM invasion. Using a 3D spheroid invasion model in collagen I matrices, we further demonstrated that KTX207 significantly suppressed TGF‑β‑induced radial invasion. Given the relatively low intrinsic invasiveness of A549 cells, TGF-β was used to induce a pro-invasive phenotype. Under these conditions, KTX207 significantly suppressed radial invasion from A549 spheroids. Because collagen I invasion depends on ITGB1‑mediated adhesion, focal adhesion signalling, and RhoA‑driven cytoskeletal remodelling, these findings reinforce a mechanistic link between PDE4D degradation and disruption of the ITGB1/FAK/RhoA axis. The attenuation of these effects by proteasome inhibition supports a degradation‑dependent mechanism.

Consistent with this, we observed disruption of tube formation and actin architecture, altered FAK localisation, as well as reduced expression of ITGB1, ezrin, VEGF-A, RhoA and ANGPT2, which are the key regulators of cell adhesion, motility, and angiogenesis, linking phenotypic changes to molecular signalling. Despite these changes, expression of canonical EMT markers, including E-cadherin, N-cadherin, SNAI1, TWIST1, Zeb1 and vimentin remained unchanged, suggesting that the phenotype is independent of EMT-related transcriptional reprogramming. These findings align with previous reports implicating PDE4 isoforms in cytoskeletal and integrin-dependent signalling [20]. Serrels et al. [21]. demonstrated that a scaffolded complex comprising FAK, RACK1, and PDE4D5 localises to nascent adhesion sites, where it regulates the initiation of cell spreading and polarity through localised cAMP signalling. This finding provides further evidence of a mechanistic link between PDE4D and integrin-FAK signalling during cytoskeletal reorganisation, bolstering the hypothesis that KTX207-mediated degradation of PDE4D could disrupt the spatial coordination of motility regulatory machinery. Additionally, the dissociation of migration speed from directional persistence underscores the role of PDE4D in directional guidance, likely mediated through RhoA-regulated cytoskeletal remodelling [22]. While the present study focused on protein expression, future investigations examining RhoA activity will provide additional mechanistic insight.

An important finding is that KTX207-mediated PDE4D degradation disrupts the spatial organisation of adhesion signalling rather than inducing global changes in kinase activation. Phospho-FAK (Y397) levels remained unchanged, whereas phospho-Src was reduced, indicating preserved FAK autophosphorylation but impaired Src-dependent amplification of focal adhesion signalling. Given the role of PDE4D in compartmentalised cAMP signalling, its degradation likely disrupts local signalling microdomains, thereby affecting Src activation and the integrity of focal adhesion complexes. Adhesion signalling is governed not only by phosphorylation status but also by precise spatial organisation, structural assembly, and coupling to the actin cytoskeleton [16]. The recruitment and retention of proteins such as FAK at adhesion complexes are essential for adhesion turnover and directional migration. Disruption of this spatial architecture is sufficient to impair cytoskeletal coordination and motility, consistent with the loss of peripheral FAK localisation following KTX207 treatment.

Defects in adhesion turnover have been linked to loss of directional migration. Ezrin, a member of the ERM (ezrin, radixin, moesin) protein family, acts as a linker between the plasma membrane and actin cytoskeleton and is critical for regulating adhesion dynamics. Overexpression of ezrin promotes invasion, whereas impaired ezrin function reduces metastatic dissemination [23–25]. Ezrin interacts with proteins such as exchange protein activated by cAMP-1 (EPAC1), potentially activating FAK independently of cell-matrix adhesion [26, 27]. Clinically, elevated ezrin expression correlates with aggressive tumour behaviour and poor patient survival [28, 29]. Although a direct PDE4D-ezrin interaction has not been demonstrated, both proteins are involved in cAMP-mediated signalling, suggesting functional crosstalk in regulating motility and invasion.

**Fig. 9:**
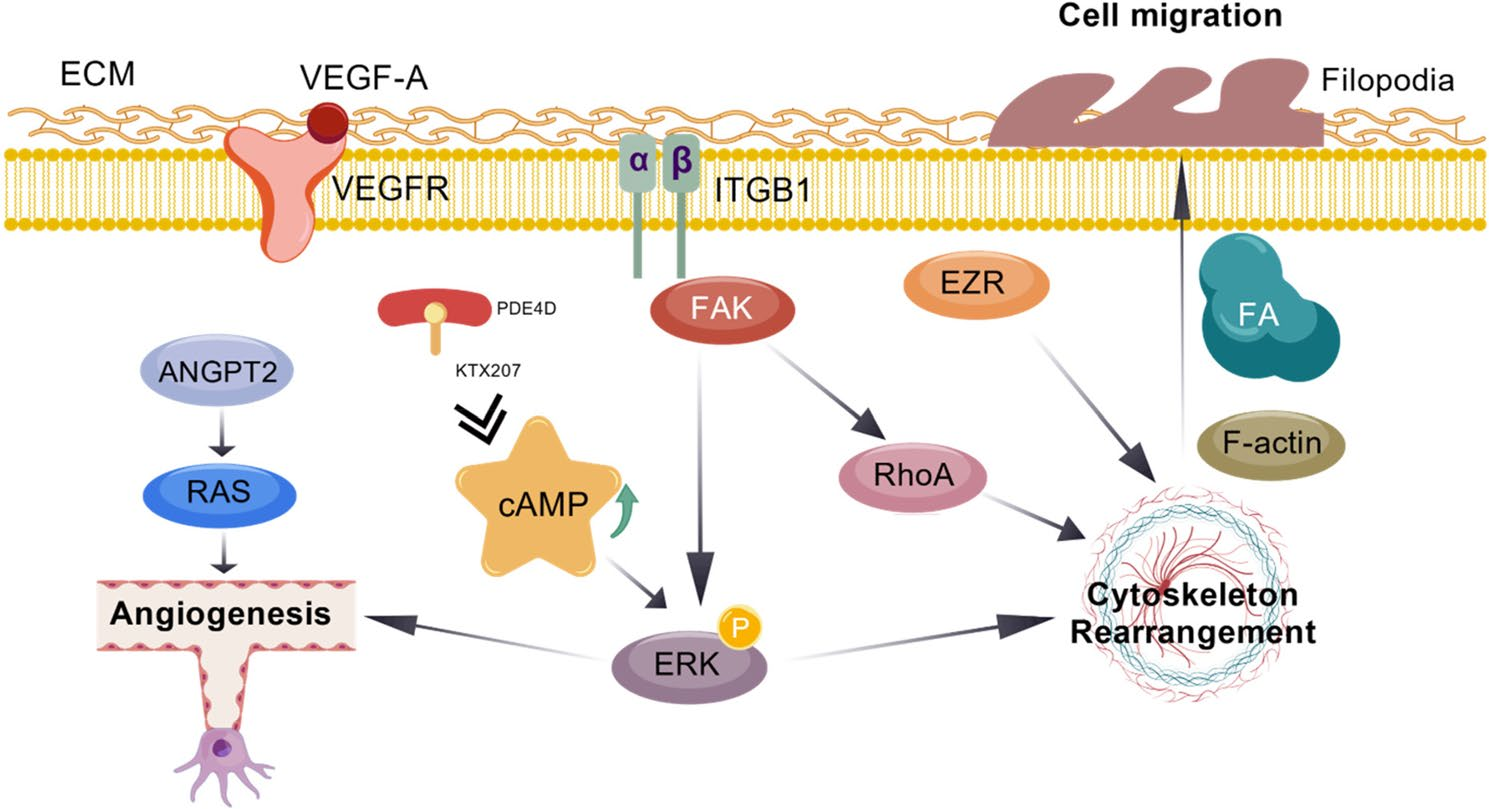
Proposed mechanism of KTX207-mediated PDE4D degradation in A549 cells. KTX207 elevates intracellular cAMP levels, reduces phospho-ERK expression, disrupts ezrin- and RhoA-actin dynamics, FAK-integrin signalling, and angiogenic mediators (VEGF-A, ANGPT2). These effects collectively lead to defective actin stress fibres, impaired focal adhesions, and loss of directional migration. The combined effects suppress cancer cell migration, cytoskeletal organisation, and angiogenesis, thereby limiting metastatic progression. EZR, ezrin. FA, focal adhesion. Created with BioGDP.com [37]

Angiogenesis is another essential step in tumour growth and dissemination. In tube formation assays, KTX207-treated endothelial cells exhibited impaired tube formation and sprouting ability, consistent with reduced VEGF-A and ANGPT2 expression. VEGF-A drives endothelial sprouting [30], while ANGPT2 regulates vascular destabilisation and remodelling [31]. To distinguish tumour‑derived from endothelial‑intrinsic effects, we performed a 3D HBEC spheroid sprouting assay with direct KTX207 treatment, which similarly showed reduced sprouting. These complementary assays indicate that KTX207 suppresses angiogenesis through both modulation of tumour‑derived VEGF and direct inhibition of endothelial behaviour. This aligns with previous studies showing that ANGPT2/ITGB1 signalling drives invasion and that blocking either ANGPT2 or ITGB1 reduces metastasis in NSCLC [32, 33]. Thus, KTX207 appears to exert dual anti-metastatic effects by suppressing both tumour cell motility and tumour-associated angiogenesis. While our functional assays provide strong evidence for impaired angiogenic behaviour, we acknowledge that expanded immunofluorescence analyses of angiogenic markers would further strengthen mechanistic interpretation and represent an important direction for future work. Notably, our previous work also showed that KTX207 reduces phospho-ERK levels [11], implicating suppression of MAPK/ERK signalling downstream of cAMP modulation. Beyond its role in proliferation, ERK regulates cytoskeletal organisation, cell polarity, and adhesion dynamics, in part by phosphorylating focal adhesion and actin-associated proteins at the leading edge of migrating cells [34]. Thus, reduced phospho-ERK likely contributes to the cytoskeletal disassembly, loss of peripheral FAK localisation, and impaired directional persistence observed in the present study. Together, these findings suggest that PDE4D shortforms function as a signalling nexus integrating cAMP and ERK pathways to coordinate cytoskeletal and angiogenic programmes that drive metastatic behaviour. Based on these findings, we propose a model to highlight the multifaceted role of PDE4D shortforms in coordinating cAMP, ERK, cytoskeletal and angiogenic signalling pathways (Fig. 9).

Previous work has shown that pharmacological PDE4 inhibition reduces cancer motility, but clinical translation has been limited by toxicity arising from poor isoform selectivity among conventional PDE4 inhibitors [35]. KTX207 is a selective degrader of PDE4D short isoforms rather than a pan-PDE inhibitor [11]. The lack of phenotypic overlap between KTX207 and the warhead control BI 1,015,550 further supports a degradation-dependent mechanism rather than off-target PDE inhibition. This PROTAC strategy offers unprecedented isoform selectivity and the potential for a more targeted PDE4D therapy with a reduced side-effect profile [36]. Nevertheless, given the structural homology within the PDE4 family, off-target degradation cannot be fully excluded. Future proteomic profiling will be required to assess target selectivity and downstream signalling specificity.

This study has several limitations. Most tumour cell-intrinsic findings were derived from a single NSCLC cell line (A549), which does not fully capture the molecular and phenotypic heterogeneity of lung cancer. We sought to address this by incorporating complementary experimental systems. Angiogenesis-related effects were evaluated using endothelial HBEC-5i cells, and functional assays were extended to 3D models, including spheroid invasion and endothelial sprouting assays, which better recapitulate aspects of the tumour microenvironment. These additional systems support the robustness and broader relevance of the observed phenotypes beyond conventional 2D cultures. Future studies should extend these findings to additional cancer cell lines with differing invasive and metastatic capacities, including more aggressive models such as small cell lung cancer (SCLC). Incorporation of advanced systems, such as patient-derived organoids, co-culture systems and ex vivo tumour explants, would further enhance physiological relevance. ITGB1 deletion has been shown to abolish FAK phosphorylation and impair lung adenocarcinoma progression [18]. Future work should therefore extend these findings to in vivo models to establish therapeutic relevance. Xenograft or orthotopic tumour systems would enable assessment of whether KTX207 suppresses primary tumour growth, limits metastatic dissemination, or impairs colonisation of distal organs. Such studies would also clarify whether the cytoskeletal and angiogenic effects observed in vitro translate into reduced tumour progression in vivo. In addition, comparative studies using non-selective PDE4 inhibitors would help determine whether isoform-selective degradation produces distinct phenotypic outcomes in whole organism models. Together, these approaches will be important for defining the broader applicability and clinical relevance of targeted PDE4D degradation in cancer.

## Conclusion

In summary, our findings demonstrate that KTX207-mediated PDE4D degradation suppresses metastatic traits, at least in part by disrupting the ezrin/ITGB1/FAK signalling axis, a central pathway governing cell migratory behaviour and cytoskeletal dynamics. Disruption of this axis compromises motility, angiogenesis, spatial organised of adhesion signalling, thereby impairing metastatic dissemination. Collectively, these results provide mechanistic insight into the role of PDE4D shortforms in cancer progression and highlight the therapeutic potential of selective PDE4D degraders as a strategy to target metastasis.

## Supplementary Information

Supplementary Material 1.

## Data Availability

The data underlying this article will be made available from the corresponding author upon reasonable request.

## Abbreviations

ANGPT2: Angiopoietin-2
BTZ: Bortezomib
DAPI: 4′,6-diamidino-2-phenylindole
DMSO: Dimethylsulfoxide
DOX: Doxorubicin
ECM: Extracellular matrix
EPAC1: Exchange protein activated by cAMP-1
EMT: Epithelial-to-mesenchymal transition
FAK: Focal adhesion kinase
GAPDH: Glyceraldehyde 3-phosphate dehydrogenase
ITGB1: Integrin β1
NSCLC: Non-small cell lung cancer
PDEs: Phosphodiesterases
PROTAC: Proteolysis targeting chimera
RhoA: Ras homolog gene family member A
SCLC: Small cell lung cancer
TBST: Tris-buffered saline with 0.1% Tween 20
TGF-β: Transforming growth factor‑beta
VEGF-A: Vascular endothelial growth factor A
