# Supplementary material for: KTX207-mediated PDE4D degradation disrupts tumour cell migration, invasion, and angiogenic potential
Source: Cell Commun Signal. 2026 May 21;24:400. doi: 10.1186/s12964-026-02946-5 (PMC13366928; doi:10.1186/s12964-026-02946-5)
Supplement: Supplementary file 1 — Supplementary Material 1. [file 12964_2026_2946_MOESM1_ESM.pdf]

Supplementary information

KTX207-Mediated PDE4D Degradation Disrupts Tumour Cell Migration, Invasion, and Angiogenic Potential

Alina Zorn[1^], Yi Zhao[1^], Aoife Giblin[1], Igor Belka[2], Eduardo Torres[2], Cathy Swindlehurst[2], Kyle Chan[2], David Stirling[2], George S. Baillie[1], Yuan Yan Sin[1#].

[1] School of Cardiovascular and Metabolic Health, University of Glasgow, Glasgow, UK G12 8QQ.

[2] Katalytic Therapeutics, 7966 Arjons Drive, Suite D, San Diego, USA 92126.

* These authors contributed equally to this work.

# To whom correspondence may be addressed. Email: angie.sin@glasgow.ac.uk

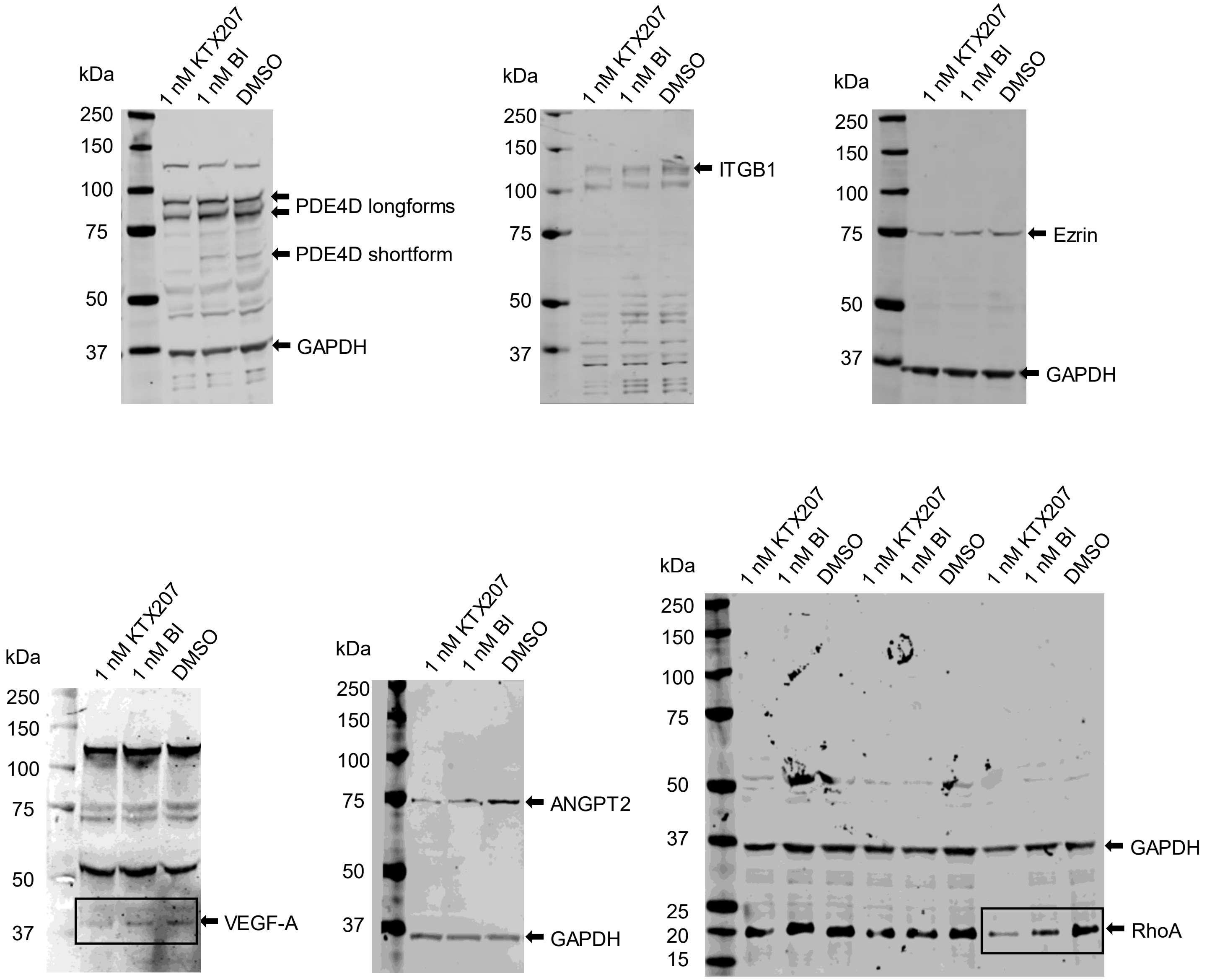

Supplementary Figure 1. Uncropped western blots of Fig. 4A.

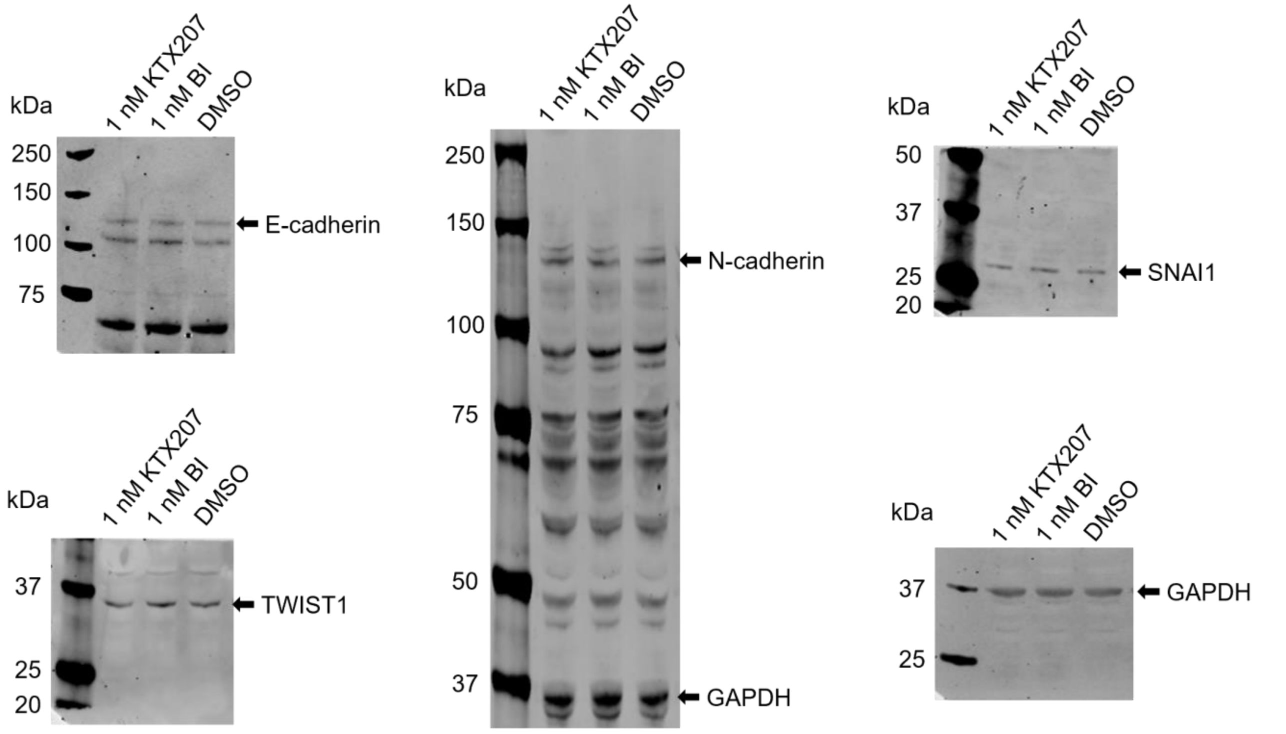

Supplementary Figure 2. Uncropped western blots of Fig. 5A.

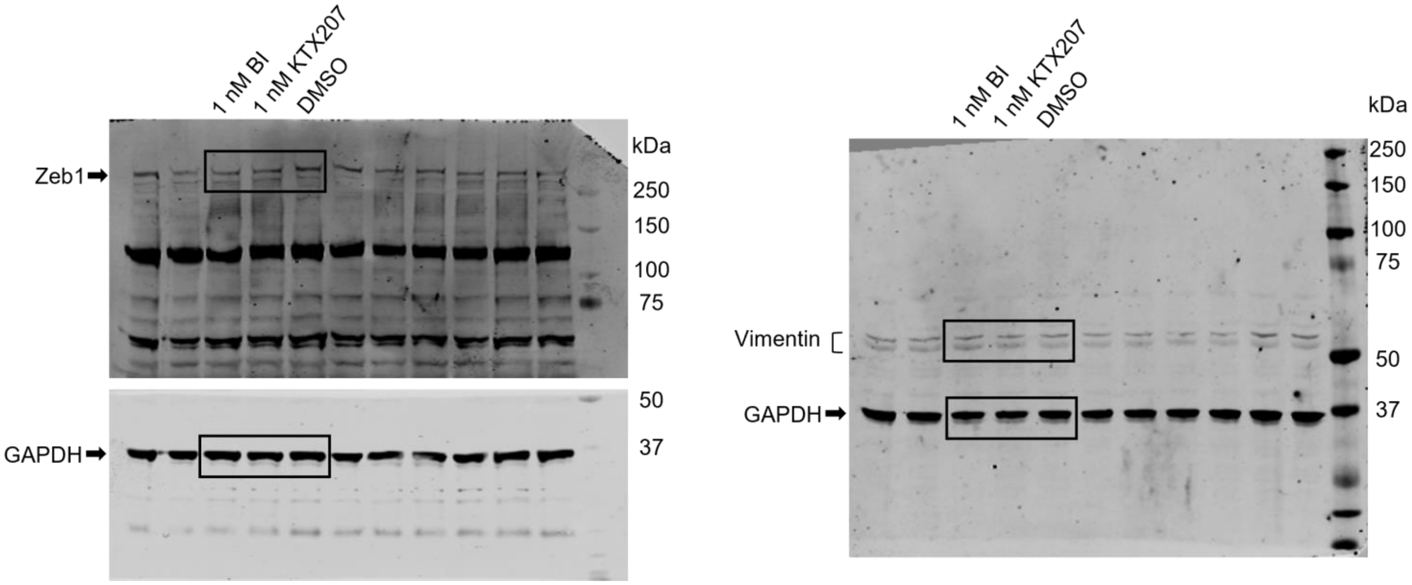

Supplementary Figure 3. Uncropped western blots of Fig. 5B.

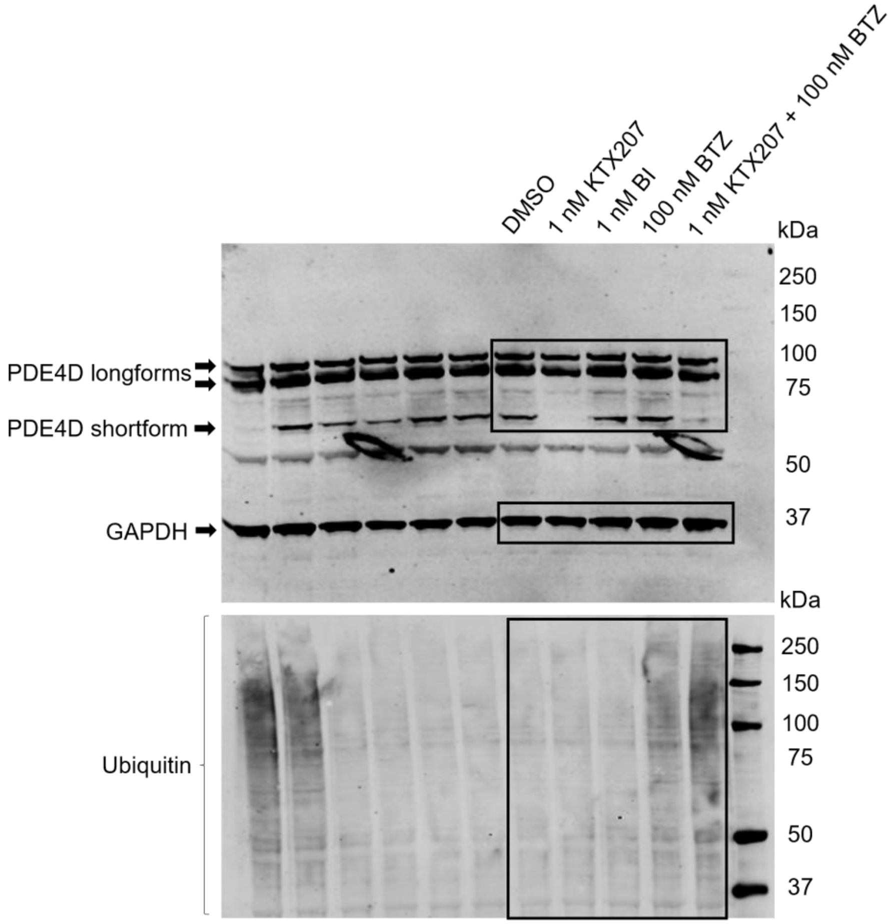

Supplementary Figure 4. Uncropped western blots of Fig. 8A.

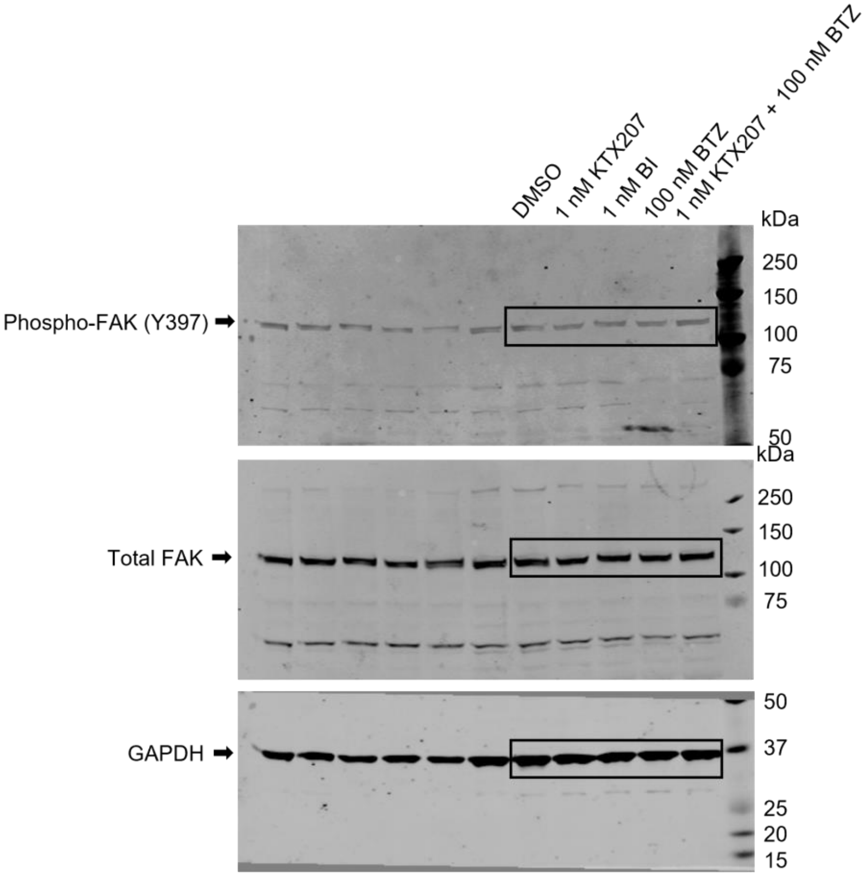

Supplementary Figure 5. Uncropped western blots of Fig. 8B.

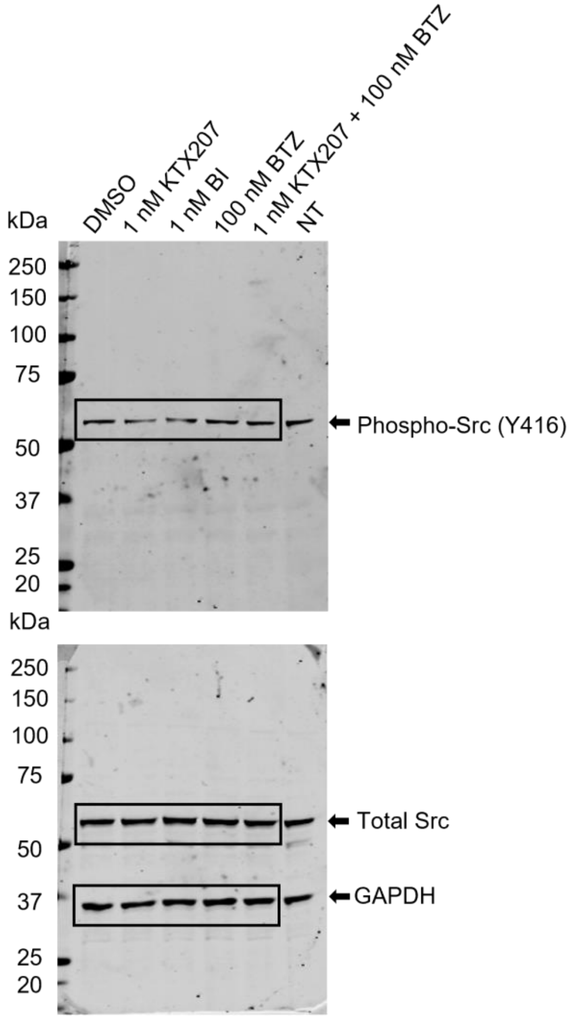

Supplementary Figure 6. Uncropped western blots of Fig. 8C.

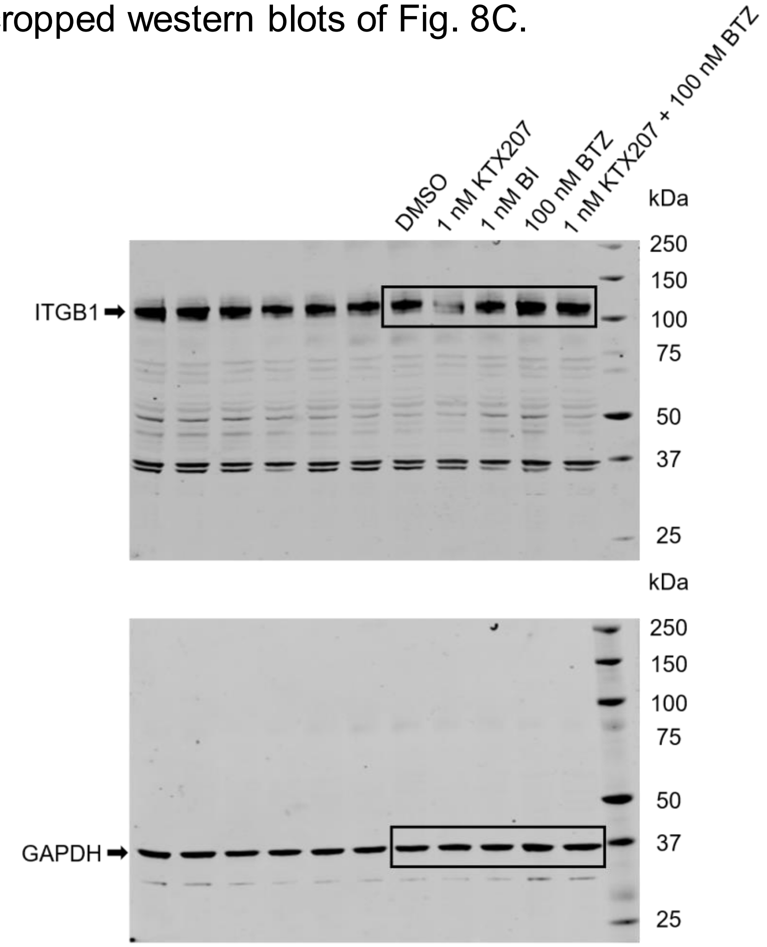

Supplementary Figure 7. Uncropped western blots of Fig. 8D.

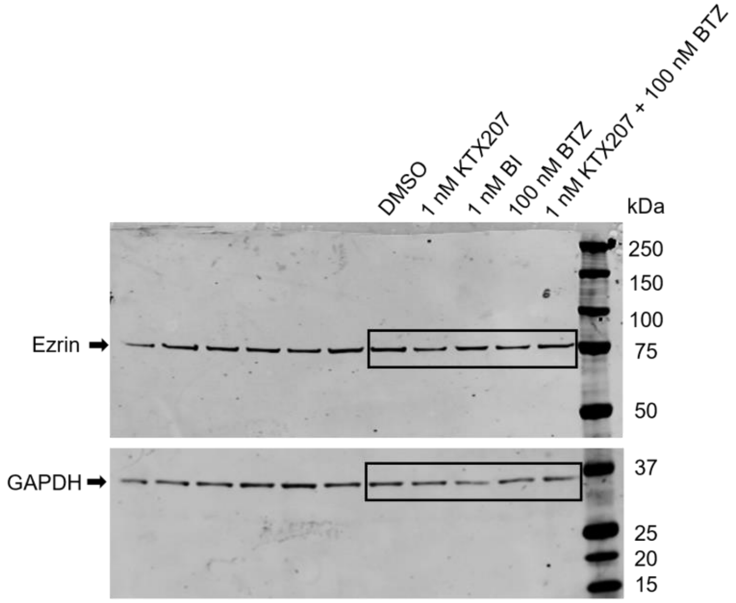

Supplementary Figure 8. Uncropped western blots of Fig. 8E.

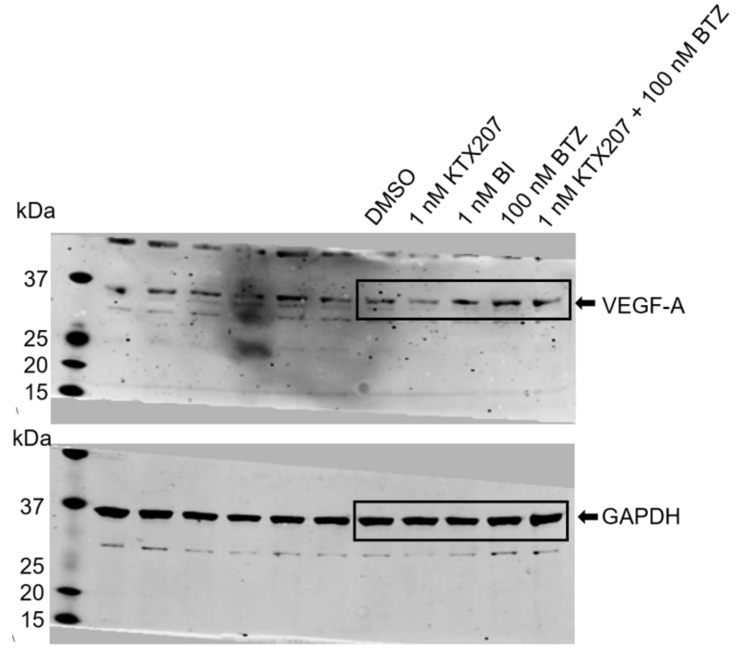

Supplementary Figure 9. Uncropped western blots of Fig. 8F.

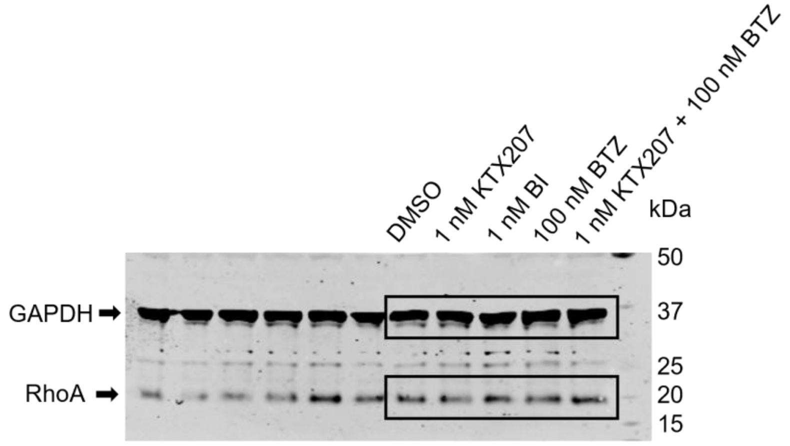

Supplementary Figure 10. Uncropped western blots of Fig. 8G.
